# DZIP3 is associated with the cytoskeletal dynamics in glioblastoma and mitochondrial machinery regulation

**DOI:** 10.3389/fbinf.2026.1878616

**Published:** 2026-10-15

**Authors:** Khadija Bichri, Imad El Badisy, Mounir Rghioui, Meryem Chihab-Eddine, Mohammed Sqalli Houssaini, Bouchra Ghazi

**Affiliations:** 1 Immunopathology-Immunotherapy-Immunomonitoring Laboratory, Faculty of Medicine, Mohammed VI University of Sciences and Health (UM6SS), Casablanca, Morocco; 2 AI and Data Science Service, Mohammed VI Center for Research and Innovation (CM6RI), Rabat, Morocco; 3 Mohammed VI University of Sciences and Health (UM6SS), Casablanca, Morocco; 4 Department of Neurosurgery, Cheikh Khalifa International University Hospital, Casablanca, Morocco; 5 Department of Biological Sciences, Faculty of Medicine, Mohammed VI University of Health Sciences, Casablanca, Morocco; 6 Department of Obstetrics and Gynecology, Mohammed VI International University Hospital, Bouskoura, Morocco; 7 Reproductive Health Physiopathology Laboratory, Mohammed IV Center for Research and Innovation (CM6RI), Rabat, Morocco

**Keywords:** DAZ interacting zinc finger 3, glioblastoma, immune microenvironment, mitochondria, RNA-binding protein, stress granules

## Abstract

Glioblastoma (GBM) is a major public health issue with increased prevalence and incidence worldwide. The therapeutic strategy is based on surgical resection, followed by chemotherapy and radiotherapy. However, therapeutic failure is increasingly recognized as driven by the intricate characteristics of the tumor microenvironment (TME). Stress granules (SGs) are structures that emerge in cells in response to stress. In GBM, these elements are implicated in tumor development and progression. Heterogeneous elements coalesce to form SGs, with RNA-binding proteins (RBPs) among them. DZIP3, DAZ-interacting zinc finger 3, is an understudied RBP in cancer, particularly with respect to its impact on TME remodeling. In the current study, we evaluate the prognostic value of DZIP3 using The Cancer Genome Atlas and the Chinese Glioma Genome Atlas as the validation cohort. Furthermore, we identified enriched pathways associated with upregulated genes linked to high DZIP3 expression. The correlation between these gene sets and DZIP3 was validated using a robustness assessment. We also identified the core hub miRNAs that target DZIP3 by using validated miRNA databases. Our results revealed that initially, high DZIP3 expression was associated with better survival in the TCGA cohort, but after adjustment for age, gender, transcription subtypes, IDH mutation status, MGMT promoter methylation status, and Karnofsky performance score, the prognostic utility of DZIP3 did not persist. Similar results were observed in the CGGA cohort. Of the 30 enriched pathways obtained, only 7 retained independent association: the cytoskeletal dynamics/intracellular transport gene set was the strongest correlate of DZIP3, followed by 4 mitochondrial modules (branched-chain amino acid metabolism, NAD biosynthesis, translation factors, and pyruvate metabolism), RNA granule/neurodegeneration gene set, and the nucleocytoplasmic transport gene set. The immune checkpoints, P-bodies, and malignant cell state gene sets were not independent of this signal. The cytoskeletal signal was replicated in the CGGA cohort and at single-cell resolution, with 9 core hub miRNAs targeting DZIP3, suspected to be a regulatory mechanism. Altogether, this study identifies the cytoskeletal module, 4 mitochondrial programs, RNA granule/neurodegeneration, and nucleocytoplasmic transport gene sets as the strongest correlates of DZIP3 expression in GBM, demonstrating that clinical prognostic value is distinct from transcriptional reproducibility.

## 1 Introduction

Despite recent advances in cancer treatment, glioblastoma (GBM) remains a public health issue, given its aggressiveness and high morbidity (Królikowska et al., 2025). Representing 45%–50% of all gliomas, the median overall survival ranges from 12 to 15 months, increasing to approximately 18 months with the current standard-of-care treatment, the STUPP protocol (Thakkar et al., 2014; Sipos et al., 2025; Loebel et al., 2025). Currently, for newly diagnosed GBM, the standard of care consists of neurosurgical resection, followed by radiotherapy concomitant to chemotherapy, and subsequent 6 months of chemotherapy alone (Fernandes et al., 2017). Molecular features of GBM that contribute to its invasive profile include mutations in the epidermal growth factor receptor and the telomerase reverse transcriptase promoter, as well as aberrant chromosomal modifications (Singh et al., 2025). The less aggressive phenotype is associated with IDH mutation status and benefits from the standard therapy in the long run (Jr et al., 2018; Malta et al., 2018).

Several therapies were developed with the hope of improving patients’ outcomes (Rios et al., 2024). Immunotherapy has emerged as a promising therapeutic strategy. Targeting inhibitory immune checkpoints (ICPs), namely programmed cell death protein 1 (PD-1) and Cytotoxic T-lymphocyte Antigen-4 (CTLA-4), has shown clinical efficacy in patients with melanoma and non-small cell lung cancer (Cho, 2017; Eggermont et al., 2016). However, in GBM, immunotherapy has yielded little improvement in overall survival (Liu et al., 2024). Immunotherapy failure results from multiple factors, including the tumor microenvironment (TME), low tumor-burden mutations, oncogenic mutations, alterations in signaling pathways, and the composition of the gut and intratumoral microbiome (Bashash et al., 2022). Cellular stress is also a culprit in immunotherapy failure (Hwang et al., 2025; Chouaib et al., 2017; Golonko et al., 2024; Wang et al., 2025). In a pan-cancer study, a new subset of T cells, T stress response cells (T_STR_), was discovered within TME. T_STR_ emerges in response to cellular stress and expresses heat shock genes, while being associated with resistance to ICP blockade (Chu et al., 2023).

Stress granules (SGs) are often depicted as reversible, membraneless structures in the cytoplasm that form in response to cellular stress (Collier and Schlesinger, 1986). They consist of aggregates of RNA-binding proteins (RBPs), translation initiation factors, non-RNA-binding proteins, ribosomal subunits, and stalled mRNAs in translation (Protter and Parker, 2016). To maintain cellular homeostasis under stressful conditions, SGs act as a temporary protective mechanism by blocking mRNA translation, thereby regulating protein synthesis (Yang et al., 2025; Huang HS. et al., 2025). Once the stress is relieved, SGs dissolve (Desai et al., 2025). However, in the case of chronic stress, SGs tend to exhibit pathological features (Protter and Parker, 2016; Jia et al., 2024). SGs are associated with different diseases, including cancer. As a matter of fact, SGs inhibit apoptosis by negatively regulating the SAPK apoptotic pathway, thereby highlighting their implication in chemoresistance (Arimoto et al., 2008). By remodulating translational processes through the PI3K/mTOR pathway, SGs can also regulate mitochondrial proteins, thereby allowing cellular stress adaptation and therapeutic resistance (Lin et al., 2024). SGs can also control key components of the antitumor immune response. Indeed, several ICPs’ mRNAs, including PD-1, were found to be regulated by SGs in cancer; once PD-1 mRNA is freed from them, it is translated and expressed on cells (Franchini et al., 2019). Despite these findings, how SGs are involved in gliomagenesis remains largely unexplored, offering an underexploited reservoir of candidate regulators.

To identify a novel, biologically interesting candidate within this compartment, we conducted a systematic literature review of SG-associated proteins to assess the extent of existing mechanistic characterization in GBM. This screening helped us identify DAZ-interacting zinc finger 3 (DZIP3), also known as hRUL138, an RBP that remains understudied despite being an E3 Ubiquitin ligase responsible for protein degradation via the proteasome (Kreft and Nassal, 2003). Previous literature indicates that DZIP3 can act as both a repressor and an activator of transcription (Zhou et al., 2008; Purcell et al., 2015). Regarding its oncogenic role, DZIP3 was found to have contradictory effects in tumors, likely context-dependent. On one hand, DZIP3 was found to promote tumorigenesis in gastric, breast, lung, colon, liver, and pancreatic cancers (Quan et al., 2020; Liang et al., 2020; Shankar et al., 2024; Jucht et al., 2025). Moreover, DZIP3 was also found to be associated with immune system aging and CD4^+^ T cells in early-stage colorectal cancer (Quan et al., 2020). On the other hand, DZIP3 exhibited antitumoral properties in low-grade glioma, where its high expression is negatively correlated with angiogenesis (Liang et al., 2020). Furthermore, DZIP3 is also a novel biomarker used for the early detection of colorectal cancer and the stratification of lower-grade wild-type IDH1 glioma (Quan et al., 2020; Liang et al., 2020). The duality of DZIP3 remains unexplored in the context of GBM.

Single-cell studies revealed the cellular heterogeneity of GBM, particularly the presence of stem-like tumor cell states (NPC-like and OPC-like) (Jucht et al., 2025). Growing evidence points to the contribution of the NPC-like phenotype to treatment resistance and tumor recurrence (Shankar et al., 2024). Moreover, stem-like states favor oxidative phosphorylation for metabolic flexibility, in contrast to the MES-like subtype, which favors glycolysis (Jucht et al., 2025). DZIP3 has previously been characterized as a transcriptional repressor by monoubiquitinating histone H2A, but it can also act as a transcriptional activator by binding CARM-1 and enhancing transcription of the estrogen receptor α (Zhou et al., 2008; Purcell et al., 2015). However, its transcriptional correlates and the cellular states associated with it in GBM remain unknown. This study aims to investigate the role of DZIP3 in GBM and its association with GBM cellular and metabolic states. We further investigated the relationship between DZIP3 and immune checkpoint expression and immune cell exclusion, and assessed its clinical significance as a prognostic marker (Figure 1). Our study presents some novelty, although it is solely based on bioinformatic tools: to characterize DZIP3 in GBM, we performed a systematic robustness assessment of 30 candidate gene sets. This analysis helped us identify independent retained transcriptional correlates. The stronger association was with the cytoskeletal dynamics/intracellular transport gene set. This correlation was replicated in an independent external validation cohort (CGGA) and at single-cell resolution. Other correlates include mitochondrial modules (branched-chain amino acid metabolism, NAD biosynthesis, translation factors, and pyruvate metabolism), RNA granule/neurodegeneration, and nucleocytoplasmic transport, and a potential miRNA-mediated control circuit associating DZIP3 to the cytoskeletal transcriptional program. Altogether, these results show that clinical prognostic functionality and transcriptional reproducibility are separate characteristics, with a relevance far beyond DZIP3 itself.

**FIGURE 1:**
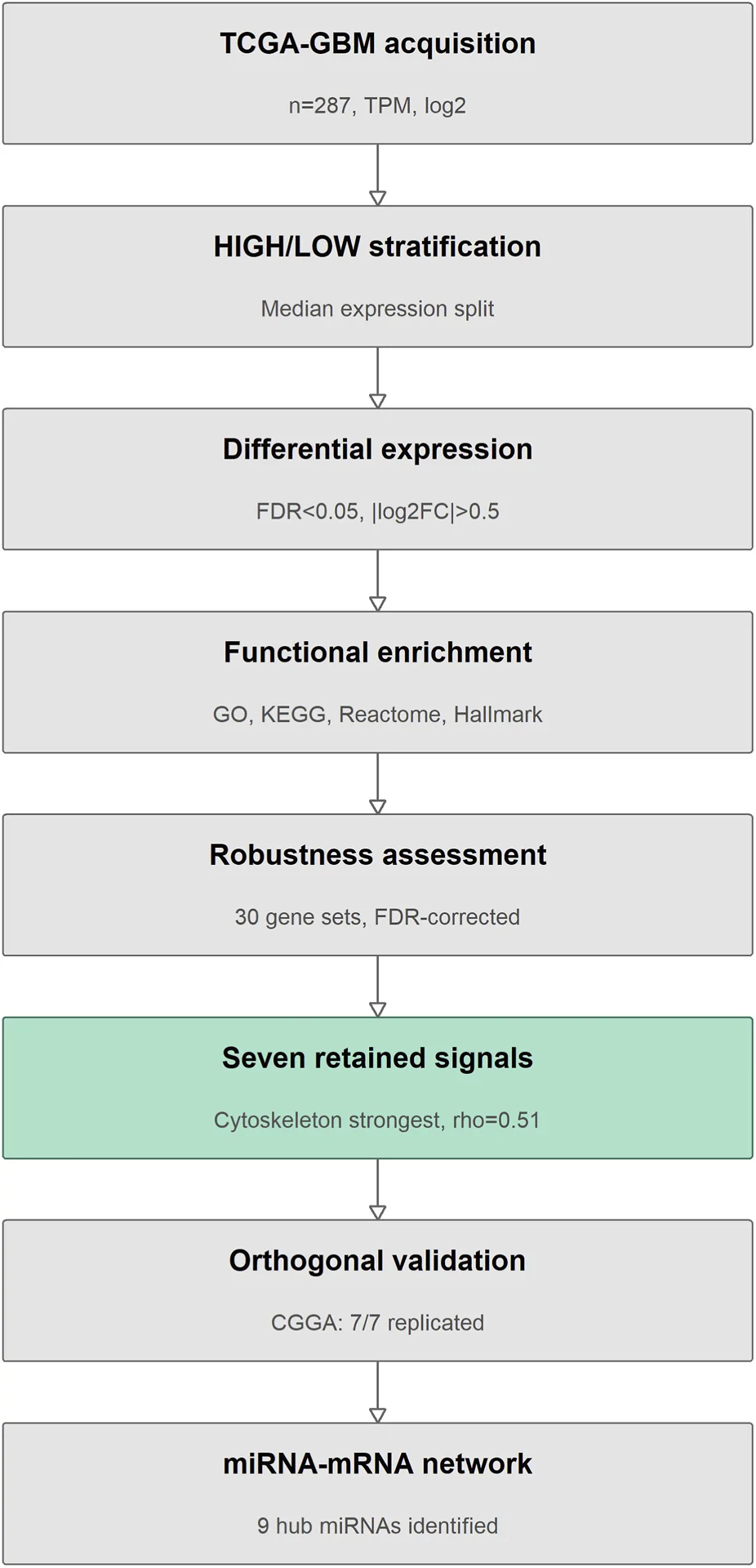
Pipeline of the analytical workflow. We used the TCGA-GBM cohort (n = 287) to investigate the characteristics of DZIP3. We dichotomized the population by median DZIP3 expression. Differential analysis and functional enrichments identified 30 gene sets. We investigated their correlation using a robustness assessment analysis, while merging FDR correction and an effect size threshold. We retained 7 gene sets, with the cytoskeletal dynamics/intracellular transport gene set being the strongest correlate of DZIP3 expression. Using the CGGA cohort for independent external validation, we find replicated signals for all the gene sets, and the stronger signal was also replicated at single-cell resolution. We also identified a miRNA-mRNA regulatory circuit associated with the gene set.

## 2 Materials and methods

### 2.1 Bulk RNA sequencing data analysis

We downloaded data from the project TCGA-GBM using the R package TCGAbiolinks version 2.35.3 (Colaprico et al., 2016). The data were derived from the STAR-count RNAseq platform. All data were raw counts, downloaded via GDquery(), GDCprepare(), and GDCdownload() functions. The conversion of raw counts to Transcripts Per Million (TPM) was based on the obtention of gene lengths (end_position–start_position +1) from Ensembl (biomaRt, hsapiens_gene_ensembl) and a standard length_normalization. TPM values were log2-transformed [log2(TPM+1)] for downstream analyses. We excluded all genes with zero expression across all samples. After matching RNA-sequence data with clinical data and removing duplicate patients, we retrieved 287 GBM samples from the TCGA-GBM project for subsequent analysis.

### 2.2 Clinical information acquisition

Using the TCGAbiolinks package, we retrieved clinical information via the GDCquery_clinic() function. Overall survival time (OS_MONTHS) was calculated from days to death (for deceased patients) or to last follow-up (for censored patients) and converted to months. Vital status was converted to binary event indicators (0 = alive, 1 = dead). Survival analyses relied only on patients with complete survival data. In addition, molecular features, namely IDH mutation status, MGMT promoter methylation status, Karnofsky performance score, and transcriptomic subtype (classical, mesenchymal, neural, and proneural), were retrieved from the curated TCGA-GBM molecular subtype annotation using the function TCGAquery_subtype (tumor = “gbm”). Given the absence of some patient information on molecular features, we performed a multivariate Cox analysis with MGMT status (n = 227/287), Karnofsky performance score (n = 217/287), transcriptomic subtype (n = 250/287), and IDH mutation status (n = 268/287) (n = 140 complete cases).

### 2.3 Differential expression genes (DEG) analysis

The dichotomization of samples into high-DZIP3 and low-DZIP3 groups was based on the median of the log2(TPM+1) values. The Limma package v3.62.2 in R was used to identify differentially expressed genes (DEGs): a linear model was fit to log2(TPM+1) values via lmFit(), and an empirical Bayes moderation of standard errors (eBayes()) was subsequently performed (Ritchie et al., 2015). Genes with Benjamini-Hochberg adjusted p-value (FDR) < 0.05 and | log2 fold-change | > 0.5 were considered significantly differently expressed (Ritchie et al., 2015). Before enrichment analyses, microRNA genes were excluded from the DEGs list to narrow our results to protein-coding regulatory mechanisms.

### 2.4 Survival analysis

To evaluate the prognostic value of DZIP3, the packages “survival” version 3.8-6 and “survminer” version 0.5.1 were used to generate the Kaplan-Meier curve and to perform Cox univariate and multivariate analyses (Therneau, 2009; Kassambara et al., 2025). Patients were stratified into high- and low-DZIP3 groups based on the median of the log2-TPM expression value. The comparison of the Kaplan-Meier survival curves was performed using the log-rank test. Using DZIP3 expression (continuous, z-score standardized) as the primary predictor, we performed univariable Cox proportional hazards regression. We later adjusted for age at diagnosis (z-score standardized), sex, MGMT promoter methylation status, Karnofsky performance score, transcriptomic subtype, and IDH mutation status (n = 140 for the complete cases) in the multivariable model. The proportional hazards assumption was verified using Schoenfeld residuals (cox.zph()). Hazard ratios (HR) with 95% confidence intervals are reported.

### 2.5 Immune deconvolution

To assess the heterogeneity and abundance of the immune cell population, we used the Cibersort algorithm, implemented via the amritr package’s source function, on log2(TPM+1) transformed expression values with the LM22 signature matrix (22 immune cell subtypes) (Chen et al., 2018). Samples were dichotomized into high and low DZIP3 expression groups (median log2(TPM+1) stratification), and comparisons between groups of estimated immune cell fractions were performed using the Wilcoxon rank-sum test with Benjamini-Hochberg correction across all cell types. For malignant cell transcriptional states, the estimation was performed using the single-cell-derived marker gene signatures implemented via the GBMDeconvoluteR gene marker set (Ajaib et al., 2023). For each cell state, a per-sample score was calculated as the mean log2(TPM+1) expression across the corresponding markers. The comparison of scores between high and low DZIP3 groups was performed using the Wilcoxon rank-sum test, and their association with DZIP3 was assessed using Spearman’s rank correlation. To rule out confounding by global tumor purity, the mean composite transcriptional state score was additionally compared between groups using an independent Student’s t-test. Using the Ajaib signature, a general immune infiltration signature, we computed gene-wise z-scores across samples restricted to signature genes, then averaged z-scores per cell population per sample (Ajaib et al., 2023). The scores were compared between high and low DZIP3 groups using the Wilcoxon rank-sum test with Benjamini-Hochberg correction. Using ImmuCellAI, we evaluated T-cell subset states enriched for high- and low-DZIP3 groups (Miao et al., 2020). The concordance between the three deconvolution algorithms was evaluated using Spearman correlation. In robustness analyses, we used the total immune infiltration as a confounder variable.

### 2.6 Enrichment analysis

Upregulated DEGs in the population with high DZIP3 expression were enriched using the Gene Ontology Biological Process (GO: BP), Molecular Function (GO: MF), Cellular Components (GO: CC), and Reactome Pathways using the packages clusterProfiler version 4.14.6 and ReactomePA version 1.50.0 (Ashburner et al., 2000; The Gene Ontology Consortium et al., 2023; Kanehisa and Goto, 2000; Yu and He, 2016; Yu et al., 2012; Croft et al., 2011). Using a hypergeometric test with Benjamini-Hochberg correction (p-value cutoff = 0.05, q-value cutoff = 0.05), we evaluated statistical significance. Gene Set Enrichment Analysis (GSEA) was also performed on the full ranked gene list (all genes ranked by limma-moderated t-statistic, in decreasing order) to identify key signaling pathways using the Molecular Signatures Database (MSigDB) Hallmark gene sets and KEGG pathways, with Benjamini-Hochberg correction (Subramanian et al., 2005). Normalized enrichment scores (NES) and adjusted p-values are reported. All the gene sets retrieved from these analyses were later subject to robustness analysis.

### 2.7 Robustness assessment of transcriptomic enrichment signatures

To separate between DZIP3-specific transcriptional associations and potential confounding due to proliferative activity, stromal/immune cell composition and technical variation in library complexity, we evaluated the correlation between module scores for candidate gene sets obtained from GO: BP, GO: MF and GO: CC enrichment analysis, ICPs expression, mitochondrial modules, DZIP3 expression and three confounder variables: a proliferation marker score (mean log2-TPM of MKI67 (standard marker of proliferation), PCNA (S-phase marker), TOP2A (S-phase/G2-phase/M-phase marker), MCM2 (DNA replication licensing factor), CCNB1 (G2/M-phase transition marker)), showing the cell-cycle-associated gene expression, total immune cell infiltration (sum of the 22 Cibersort LM22 cell fractions) for tumor purity, and number of genes detected per sample representing the library complexity (number of genes with TPM >1 per sample), showing the technical variation in RNA quality and sequencing depth, the latter included following the observation of a significant difference in detected gene number between high and low DZIP3 expression groups (Wilcoxon p < 2.2 × 10^−16^). For the 30 gene sets evaluated, we applied the Benjamini-Hochberg correction to all partial correlation p-values, yielding FDR-adjusted p-values (padj). To control for all confounders simultaneously, we performed partial Spearman correlation using the ppcor R package when a candidate gene set score showed a correlation with DZIP3 exceeding ρ = 0.3 (ppcor, 2015). We retained gene sets as an independent signal only if both of the following criteria were met: statistical significance after FDR correction (padj <0.05) and a minimum effect size threshold of partial ρ ≥ 0.30 in absolute value, as per Cohen’s conventional benchmark for moderate effect size (Cohen, 2013). The goal of this was to determine if the associations with DZIP3 were independent or had a common underlying transcriptional signature. Gene sets that showed a statistically significant partial correlation with DZIP3 after this control were used for further biological interpretation. Moreover, the establishment of the cytoskeletal dynamics/intracellular transport was based on the enrichment of the terms retrieved from the GO: CC (axon cytoplasm, growth cone, kinesin complex) and Reactome pathways linked to centrosome organization and mitotic spindle (centrosome maturation, M phase, RHO GTPase effectors). Both terms had overlapping genes. As such, the 17 gene set obtained encompassed kinesin family motor proteins, stathmins, cytoplasmic dynein heavy chain, dynein regulator factor PAFAH1B1, and microtubule-associated proteins. This gene set is functionally associated with microtubule-dependent intracellular transport.

### 2.8 Single-cell RNA sequencing validation

Using the Core GBmap harmonized single-cell reference atlas for GBM (338564 GBM tumor cells), we performed single-cell validation (Ruiz-Moreno et al., 2025). We filtered the cells to obtain only malignant cells, using the “annotation-level-1” metadata field (“Neoplastic”, n = 127521 cells). We extracted raw count data from the sparse CSR-encoded expression matrix (H5AD format). We used HDF5 indexing to obtain expression values for DZIP3 and components of the cytoskeletal dynamics/intracellular transport gene set. We determined the Spearman correlation with and without partial correlation control for per-cell sequencing depth. As we observed different scales of correlation magnitudes between single-cell and bulk transcriptomic data, we did not apply the effect size threshold used for the bulk robustness assessment (partial ρ ≥ 0.30). The single-cell validation was evaluated solely for statistical significance and concordance with the bulk analysis, consistent with its orthogonal confirmatory role.

### 2.9 External validation using the CGGA cohort

We used the CGGA mRNAseq-693 dataset, retrieving only primary GBM histology (n = 133 after excluding samples with missing survival data) (Zhao et al., 2021). The expression values initially in FPKM were converted to log2(x+1). We performed univariate and multivariate Cox proportional hazards analyses using age, sex, IDH mutation status, MGMT promoter methylation status, and treatment status (n = 108 for complete cases). The CGGA cohort lacked the Karnofsky performance score and transcriptional subtypes.

### 2.10 Correlation with inhibiting ICPs

To assess the correlation between DZIP3 and ICPs, we compiled a list of 49 inhibitory ICPs from established literature. For each checkpoint gene, we calculated Spearman’s rank correlation between log2-TPM expression and DZIP3 log2-TPM expression. To visualize group-level differences, we separately calculated the mean expression per checkpoint in the high- and low-DZIP3 groups and evaluated group differences using the Wilcoxon rank-sum test. We used the “stats” package (version 4.4.1) to compute Spearman’s r. P-values were adjusted using the Benjamini-Hochberg procedure to control the false discovery rate (FDR). An FDR <0.05 was considered statistically significant. To evaluate whether the correlation between DZIP3 and the ICPs was not due to confounding from shared transcriptional programs, we performed a robustness assessment as described previously.

### 2.11 Correlation with mitochondrial modules

We first downloaded mitochondrial genes (1136 genes) and their corresponding functional pathway annotation from the MitoCarta 3.0 database (Rath et al., 2021). Next, the expression matrix was aligned with mitochondrial gene symbols present in both datasets. We calculated the composite mitochondrial activity score per sample as the mean log2-TPM across all mitochondrial genes and compared the scores between high- and low-DZIP3 groups using the Wilcoxon rank-sum test. Genes were clustered into MitoCarta3.0-defined functional pathways. As such, we obtained 51 functional modules. A per-sample module score was calculated as the mean linear TPM expression across constituent genes for each module containing ≥5 detected genes. The comparison of module scores between high- and low-DZIP3 groups was performed using the Wilcoxon rank-sum test. The association of module scores with DZIP3 was assessed using Spearman’s rank correlation. P-values across all modules were adjusted for multiple testing using the Benjamini-Hochberg procedure. We performed a differential analysis comparing the high- and low-DZIP3 groups using the Limma package. The upregulated DEGs (Benjamini-Hochberg-adjusted p-value (FDR) < 0.05 and |log2 fold-change| > 0.5) helped identify which mitochondrial modules DZIP3 was associated with.

### 2.12 Construction of the miRNA-mRNA network

Using the TCGAbiolinks package, we retrieved miRNA-seq data for 224 of 287 patients in the initial cohort. Using the Limma package, we identified differentially expressed miRNAs between high- and low-DZIP3 groups (FDR <0.05 and |log2FC| > 0.5). We mapped precursor-level TCGA miRNA identifiers to mature miRNA nomenclature for consistency with target databases. Using the multiMiR package, we identified miRNAs that interact with DZIP3 and with the cytoskeletal dynamics/intracellular transport gene set (Ru et al., 2014). To obtain the final network, we used experimentally validated interactions from miRTarBase, TarBase, and miRecords. We also queried computationally predicted interactions from TargetScan, miRDB, and PicTar (limited to those present in the consensus of≥2 of the 3 databases). Still, we did not integrate them into the final construct given the number of candidate interactions and their lower confidence relative to experimentally validated data. We cross-referenced the differentially expressed miRNAs against the validation interaction set. From this subset, we obtained miRNAs that target ≥13 cytoskeletal genes and were considered core hub regulators. Using the same validated database, we evaluated the direct targeting of DZIP3 by these hub miRNAs.

### 2.13 Statistical analysis

R language v4.4.1 was used for statistical analysis and graph generation. The Wilcoxon rank-sum test was performed to compare non-normally distributed continuous variables. Spearman’s rank correlation coefficient (ρ) was used to assess the association between continuous variables. The Benjamini-Hochberg procedure was applied for multiple testing correction. All statistical tests were two-sided, and statistical significance was set at FDR/adjusted p < 0.05.

## 3 Results

### 3.1 DZIP3 is not associated with prognosis in TCGA-GBM patients nor in the CGGA cohort

Using the packages “survival” and “survminer”, we generated the Kaplan-Meier curve to evaluate the impact of DZIP3 expression on patients’ survival (Figure 2). High DZIP3 expression was associated with prolonged overall survival compared with patients with low DZIP3 expression (p < 0.05). Next, we inquired whether DZIP3 expression could influence GBM prognosis. Thus, we conducted a univariate Cox proportional hazards regression analysis (Table 1). We observed that HR < 1, indicating that higher DZIP3 expression is associated with a better prognosis, although the effect is not statistically significant.

**FIGURE 2:**
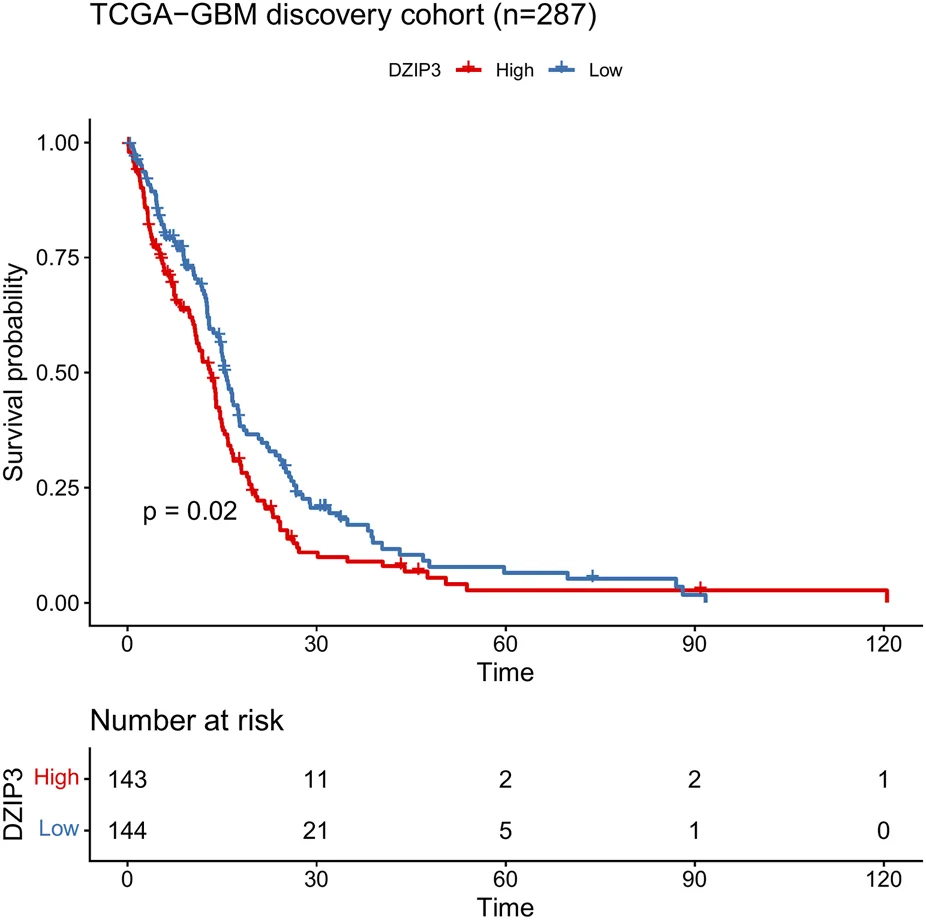
Overall survival relative to DZIP3 expression in the TCGA-GBM cohort. Kaplan-Meier survival curves comparing patients with high and low dzip3 expression (median dichotomization; n = 287). High DZIP3 expression is significantly and positively correlated with improved survival. However, this association was not independent of other clinical covariates analyzed in the multivariable analysis.

**TABLE 1:** Univariate Cox analysis for overall survival in the TCGA cohort. Univariate Cox analysis shows that DZIP3 is associated with a good prognosis, although the p-value is not significant.

Characteristic	HR	95% IC	p-value
DZIP3 expression (per 1 SD increase)	0.801	0.637–1.009	0.0592

To assess whether the expression of DZIP3 can potentially be used as a prognostic factor, independent of other variables, namely age, gender, IDH mutation status, MGMT promoter methylation, Karnofsky performance score, and transcriptomic subtype, a multivariate Cox analysis was performed (n = 140 complete cases, Table 2; Figure 3; Supplementary Table 1). The results confirm the previous outcomes of the univariate Cox analysis: DZIP3 is not significantly associated with survival (HR = 1.08, 95%CI:0.75–1.6, p = 0.66). Moreover, age, gender, and Karnofsky performance score remained significantly associated (HR = 1.02, p = 0.007; HR = 1.75, p = 0.012; and HR = 0.98, p = 0.034, respectively). On the other hand, molecular features, including IDH mutation status and MGMT promoter methylation status, showed no statistical significance (HR = 0.96, p = 0.95 and HR = 0.70, p = 0.086, respectively). This could be due to an association with other included covariates and to diminished statistical power in the complete-case subset (n = 140). To validate these findings, we replicated the same procedure in the CGGA cohort (n = 108 complete cases, Figure 4). DZIP3 is not an independent prognostic factor after adjustment for age, gender, IDH mutation status, MGMT promoter methylation, or treatment status (radiotherapy and chemotherapy) (HR = 0.87, 95%IC: 0.70–1.1, p = 0.23, Table 4; Figure 5; Supplementary Table 1). However, IDH mutant status and chemotherapy showed a strong association with survival (HR = 0.42, 95% CI: 0.221–0.798, p = 0.008 and HR = 0.33, 95% CI:0.18–0.60, p = 0.0003, respectively). Age showed statistical significance (p = 0.029), whereas MGMT promoter methylation status, radiotherapy, and gender did not. Altogether, these results show that the initial prognostic value of DZIP3 was attributable to confounding by clinical and molecular risk factors rather than to an independent biomarker effect.

**TABLE 2:** Multivariate Cox analysis for the expression of DZIP3 in z-score, age, gender, IDH mutation status, Karnofsky performance score, transcriptional subtypes, and MGMT promoter methylation status (TCGA cohort).

Characteristic	Overall survival		
	HR	95% IC	p-value
DZIP3 expression	1.085	0.753–1.564	0.662
Age at diagnosis (per 1 SD increase)	1.024	1.006–1.042	0.00735
Sex			
Female	_	_	
Male	1.749	1.133–2.701	0.0116
IDH mutation status			
WT	_	_	
Mutant	0.961	0.278–3.326	0.95
Mesenchymal subtype	1.138	0.721–1.796	0.578
Neural subtype	1.393	0.664–2.922	0.38
Proneural subtype	0.911	0.501–1.656	0.76
Karnofsky performance score	0.982	0.966–0.999	0.0344
MGMT promoter methylation status			
WT	_	_	
Methylated	0.702	0.469–1.051	0.0857

**FIGURE 3:**
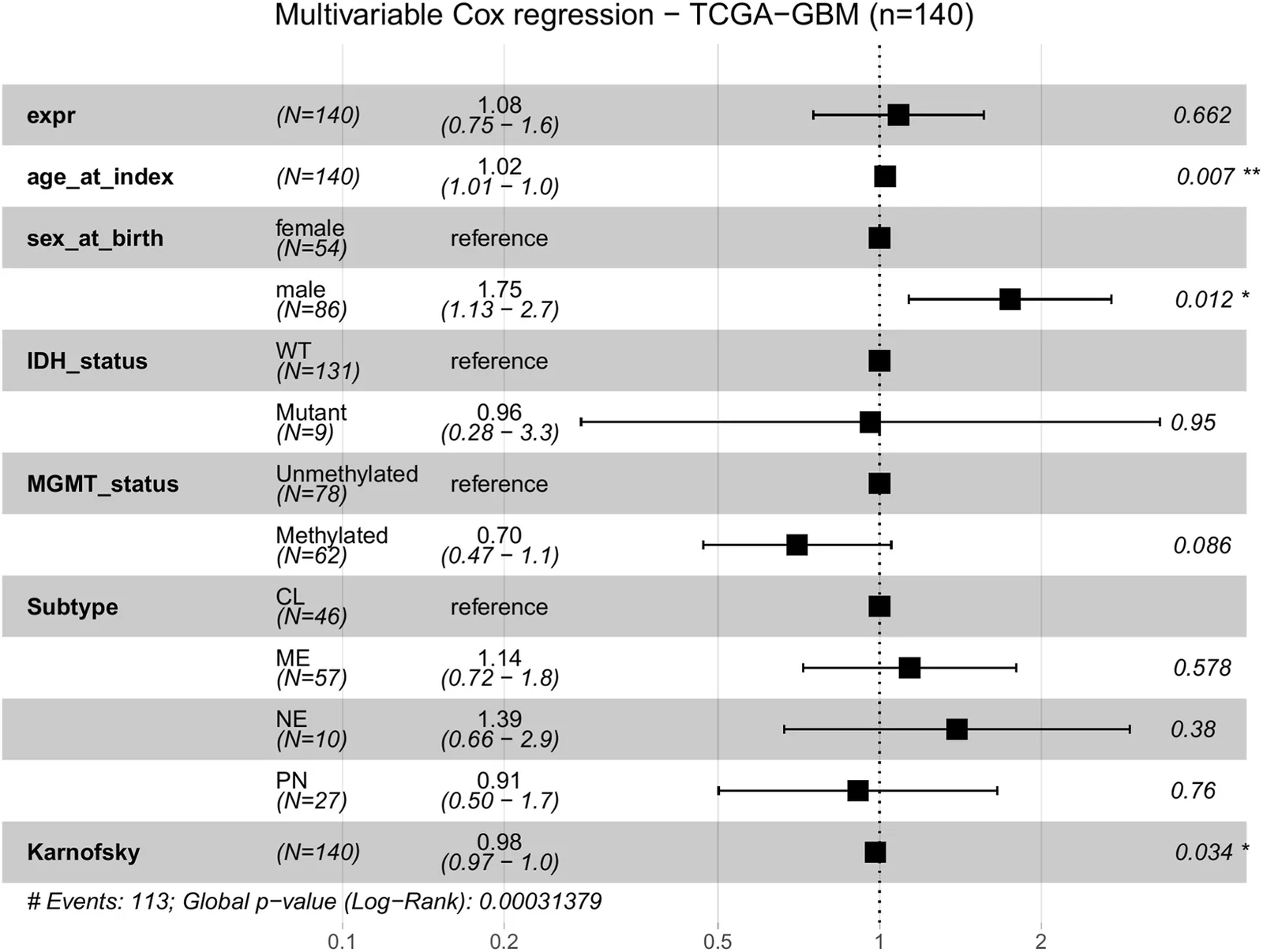
Forest Plot of Multivariate Cox analysis incorporating age, sex, IDH mutation status, MGMT promoter methylation status, transcription subtypes, and Karnofsky performance score (TCGA, n = 140).

**FIGURE 4:**
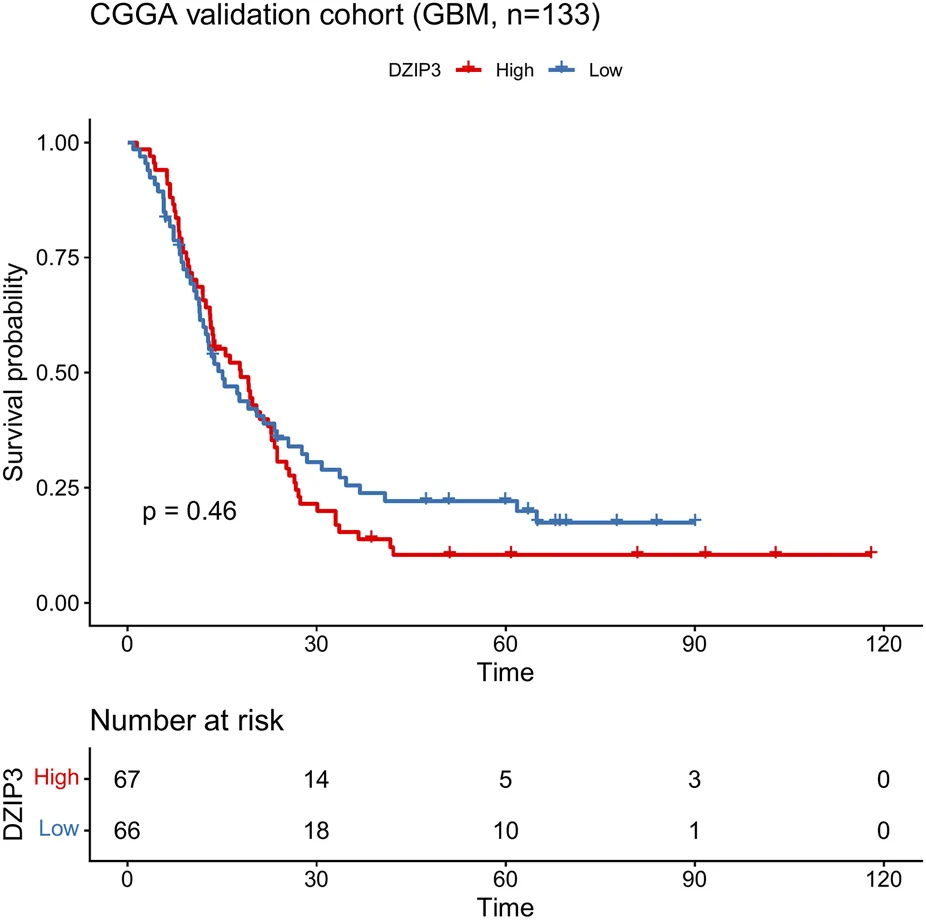
Overall survival relative to DZIP3 expression in the CGGA cohort. Unlike the TCGA GBM cohort, DZIP3 was not associated with improved survival, and no statistical significance was assessed. These results were further corroborated when performing the Cox multivariate analysis.

**TABLE 3:** Univariate Cox analysis for overall survival in the CGGA cohort. Univariate Cox analysis shows that DZIP3 is not associated with prognosis.

Characteristic	HR	95% IC	p-value
DZIP3 expression (per 1 SD increase)	0.99	0.817–1.2	0.918

**TABLE 4:** Multivariate Cox analysis for the expression of DZIP3 in z-score, age, gender, IDH mutation status, MGMT promoter methylation status and treatment status (CGGA cohort).

Characteristic	Overall survival		
	HR	95% IC	p-value
DZIP3 expression	0.874	0.701–1.091	0.234
Age at diagnosis (per 1 SD increase)	1.019	1.002–1.036	0.0289
Sex			
Female	_	_	
Male	0.98	0.619–1.551	0.931
IDH mutation status			
WT	_	_	
Mutant	0.42	0.221–0.798	0.00807
MGMT promoter methylation status			
Unmethylated	_	_	
Methylated	0.982	0.618–1.562	0.94
Therapy			
Radiotherapy	0.675	0.344–1.323	0.252
Chemotherapy	0.325	0.176–0.6	0.000323

**FIGURE 5:**
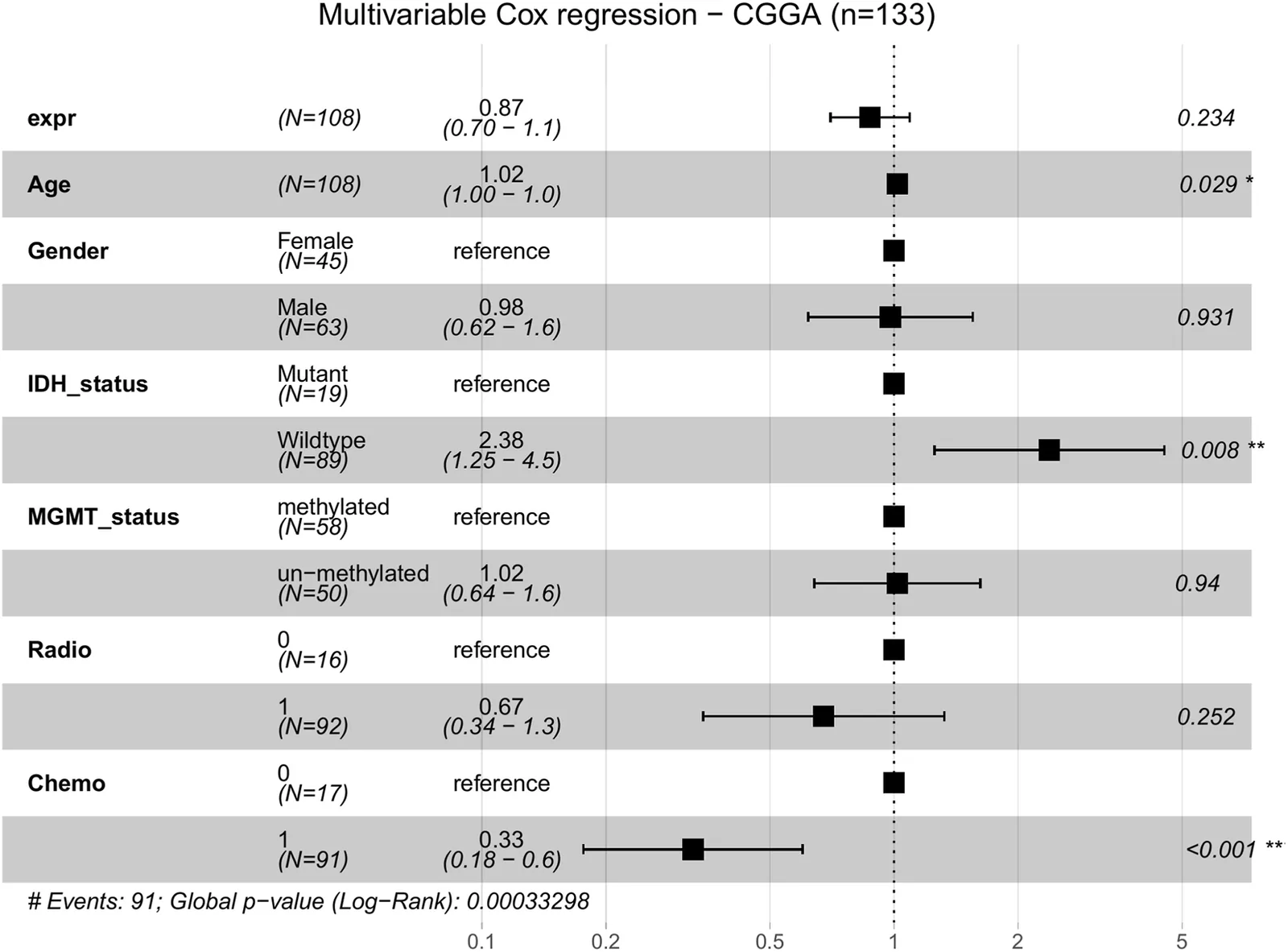
Forest Plot of Multivariate Cox analysis incorporating age, sex, IDH mutation status, MGMT promoter methylation status, and treatment status (CGGA, n = 108).

### 3.2 High DZIP3 is associated with RNA processing and neuronal development

Based on DZIP3 expression, we stratified the cohort into high- and low-DZIP3 groups using the median log2(TPM+1) as the cutoff. By exploring 38218 genes, we performed a differential analysis using Limma with empirical Bayes moderation. We identified 4028 upregulated and 364 downregulated genes (FDR <0.05, |log2FC| > 0.5; Figure 6; Supplementary Table 2). The top 20 upregulated genes are SCARNA7, SCARNA21, MIR9-1, MIR125B1, RNY1, RN7SKP255, SNORD74B, MIR4803, RNY3P1, RNU6-130P, RN7SKP80, H1-4, RNU4-88P, RN7SKP71, RNU5D-1, RNU5B-1, H4C5, RN7SKP9, H4C3 and MIR3609 (Supplementary Table 3). These genes are associated with neurodevelopmental properties, RNA processing, tumor suppressors, tumor enhancers, or biomarker roles (Nava et al., 2025; Schattner et al., 2006; Jia et al., 2021; Wang et al., 2020; Reichart et al., 2025; Taleb et al., 2025; Abasi et al., 2016; Xu et al., 2017; Chen et al., 2025; Yuan et al., 2025). These results confirm that genes associated with DZIP3 are consistent with previous literature on DZIP3’s role in neurodevelopment (Quan et al., 2020; Liang et al., 2020; Inoue et al., 2015; Kolapalli et al., 2021). The top 20 downregulated genes are MT-ATP8, MTATP6P1, MT-ND1, MT-ND6, MT-ND2, MT-ATP6, MT2P1, PLA2G2A, MT-ND3, MTND2P28, MT-ND4, C6orf15, CHI3L1, SAA1, MT-ND4L, MT-CYB, PI3, SLPI, MTND1P23 and MTATP8P1 (Supplementary Table 4). Interestingly, low-expressed genes are associated with oxidative phosphorylation (OXPHOS), extracellular matrix composition, inflammation and cancer remodeling, fatty acid metabolism, wound healing, and anti-inflammatory properties (Ganetzky et al., 2019; Yu J. et al., 2024; Yu JE. et al., 2024; Li et al., 2017; Zhang et al., 2022; Nugteren and Samsom, 2021).

**FIGURE 6:**
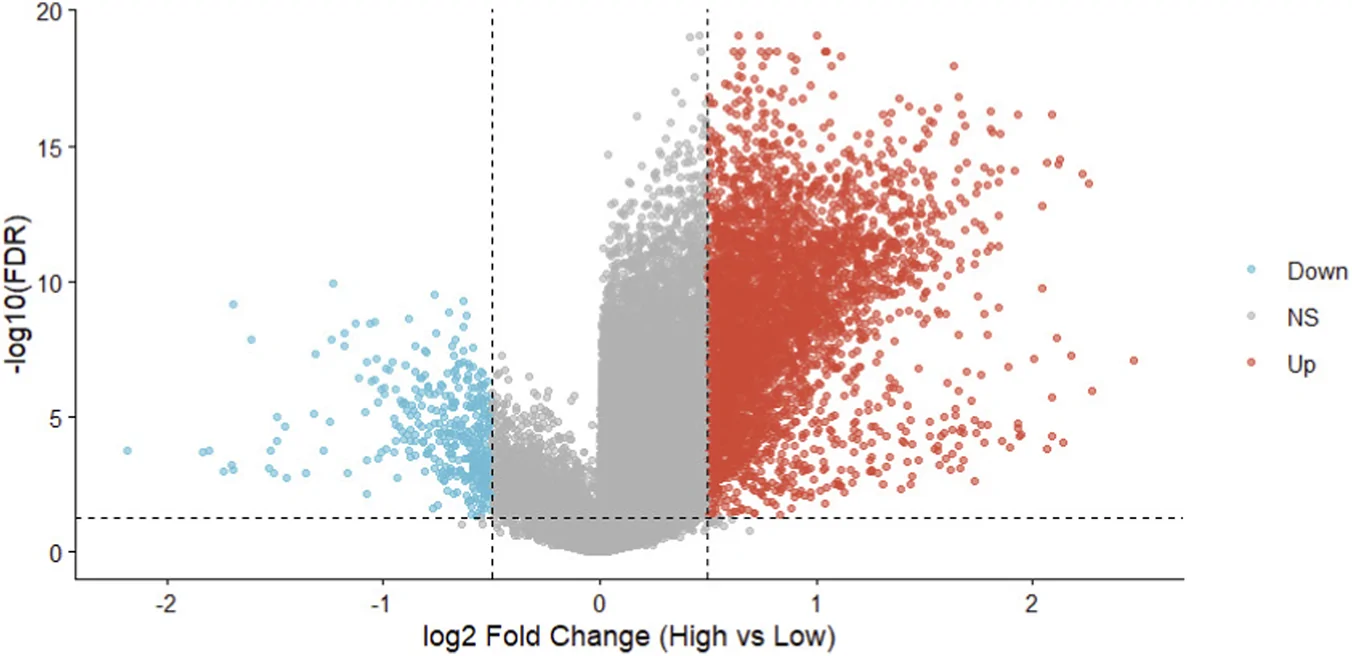
Differential gene analysis performed between high and low DZIP3 groups. Volcano plot of limma differential expression genes (n = 287). Genes upregulated (FDR <0.05 and log2FC > 0.5) and downregulated (FDR <0.05 and log2FC < -0.5) are respectively highlighted in red and blue.

### 3.3 DZIP3 associates mainly with the cytoskeletal dynamics/intracellular transport gene set

After filtering the ranked gene lists from the Limma differential analysis (FDR <0.05 and |log2FC| > 0.5) to exclude microRNAs, we performed Gene Ontology (GO) enrichment analysis. GO terms related to biological processes (GO: BP) revealed that upregulated genes in the high DZIP3 group are enriched in 111 significantly enriched terms (Supplementary Table 5). These terms were mainly associated with nucleosome organization, spliceosomal snRNP assembly, glial/oligodendrocyte differentiation, humoral immune response, cytoskeletal transport, and metal ion homeostasis (Figure 7a).

**FIGURE 7:**
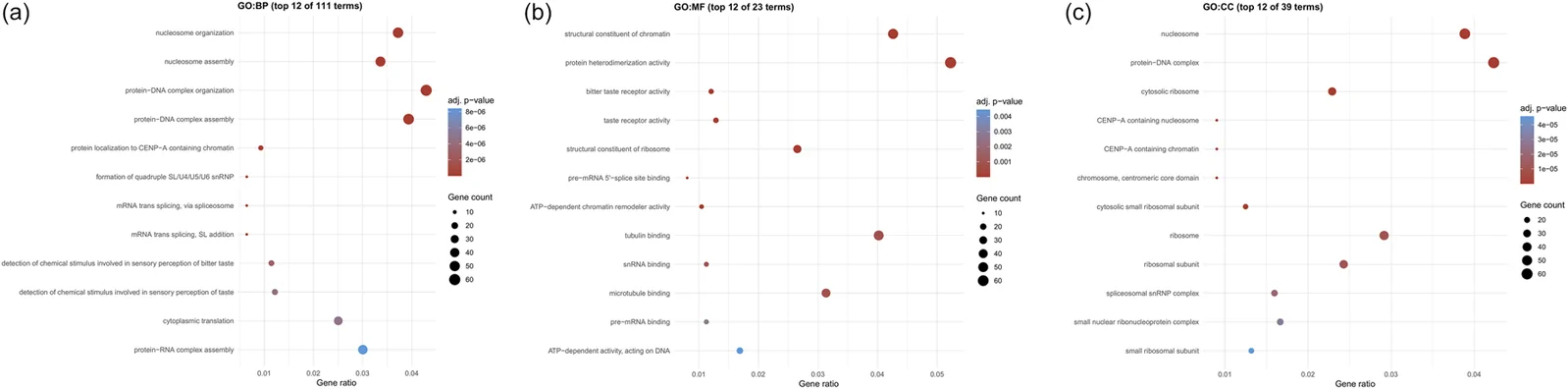
Gene Ontology enrichment analysis performed on upregulated genes in high DZIP3 tumors. The enriched terms are shown across (FDR <0.05): **(a)** Biological Process: identification of 111 significantly enriched processes; **(b)** Molecular Function: identification of 23 enriched terms; **(c)** Cellular Component: identification of 39 significantly enriched terms. We limited the number of terms shown to 12 per section, using an adjusted p-value (Benjamini-Hochberg) for readability. All the results are in the supplementary table. The enrichments reflect statistically significant correlations prior to the robustness assessment.

On the other hand, the GO enrichment on molecular functions (GO: MF) identified 23 significantly enriched terms (Supplementary Table 6), which are related to structural constituent of chromatin, protein heterodimerization activity, structural constituent of ribosome, pre-mRNA/snRNA binding, ATP-dependent chromatin remodeler activity, tubulin/microtubule binding, histone binding, kinase regulator activity, CXCR chemokine receptor binding and histone H3K36 methyltransferase activity (GO:0030527, GO:0046982) (Figure 7b).

Additionally, the GO enrichment analysis of cellular components (GO: CC) identified 39 significantly enriched terms (Supplementary Table 7). We identified terms associated with nucleosome and protein-DNA complex terms, ribosomal and spliceosomal snRNP complex terms, chromosomal region terms, cytoplasmic-ribonucleoprotein granule/P-body terms, and axon/cytoskeleton related terms (Figure 7c).

### 3.4 Reactome ORA enrichment

Next, we investigated biological pathways associated with high DZIP3 (Figure 8). We identified 190 significantly enriched pathways (padj <0.05, Supplementary Table 8). Most of them were already characterized in the GO enrichments (ribosomal/translation genes, histones, metallothioneins, P-bodies, chromatin-modifying enzymes, and glial lineage signature). The cytoskeletal dynamics/transport gene sets overlapped with pathways related to mitotic spindle and centrosome organization. We also identified the NOTCH signaling pathway (pre-NOTCH transcription and translation, NOTCH signaling) and WNT signaling (WNT signaling and TCF-dependent signaling in response to WNT).

**FIGURE 8:**
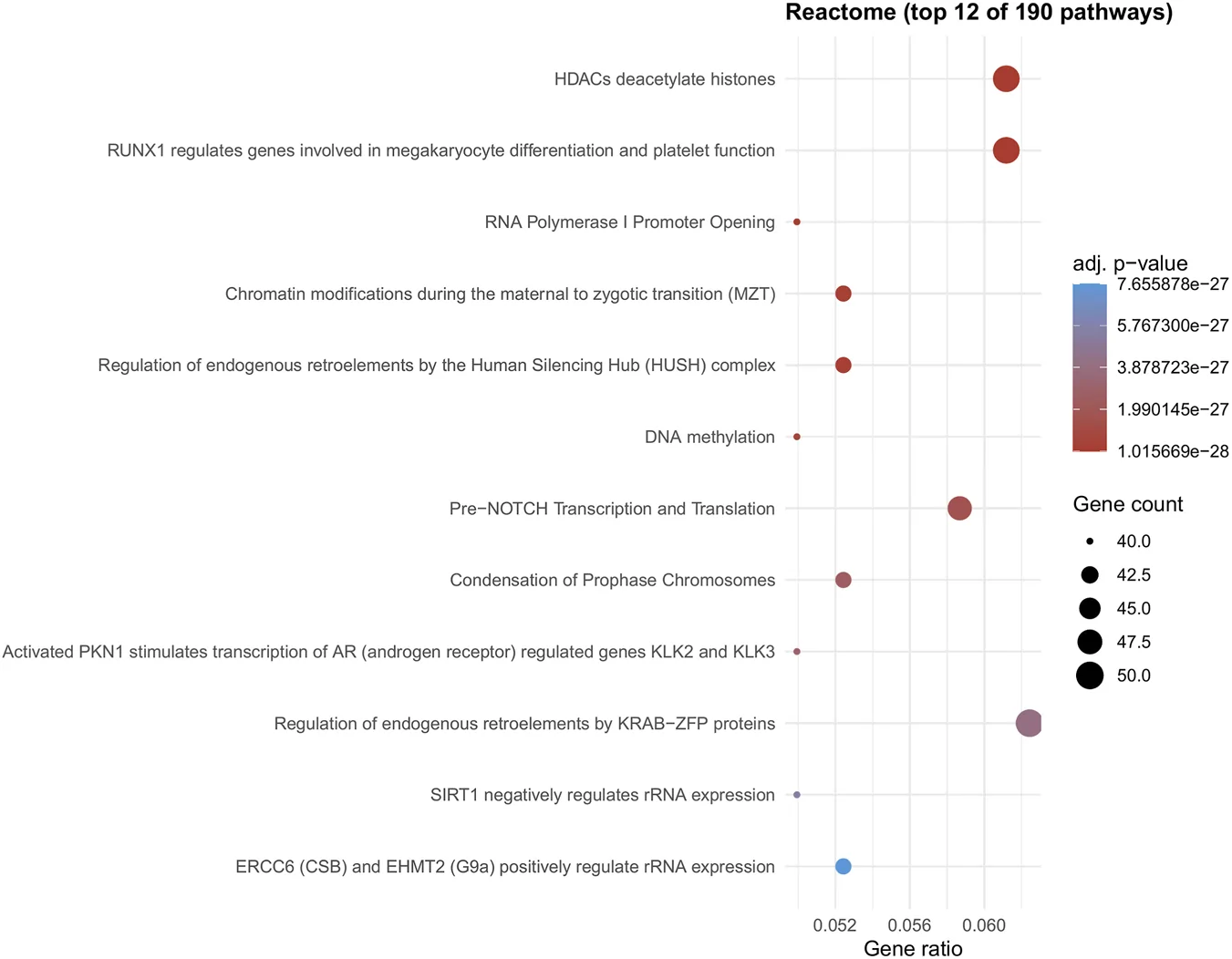
Reactome analysis identified 190 significantly enriched terms associated with ribosomal/translation genes, histones, metallothioneins, P-bodies, chromatin modifying enzymes, and glial lineage signature. The top terms are shown by gene count and adjusted p-value (FDR < 0.05). The enrichments reflect statistically significant correlations prior to the robustness assessment.

### 3.5 KEGG GSEA enrichment

The ranked gene list obtained from the Limma analysis was used to perform a GSEA using the KEGG pathways (Figure 9). We identified 224 significantly enriched pathways (padj <0.05, Supplementary Table 9). RNA processing, cell cycle, ubiquitination, and immune signaling pathways (TNF signaling, TLR signaling, chemokine receptor signaling, PD-1 and PD-L1 checkpoint) were among these subsets. Another interesting subset related to diseases was retrieved (neurodegenerative diseases, cancer) and had common gene content (oxidative phosphorylation, proteasome, ribosomal proteins) rather than being independent biological processes.

**FIGURE 9:**
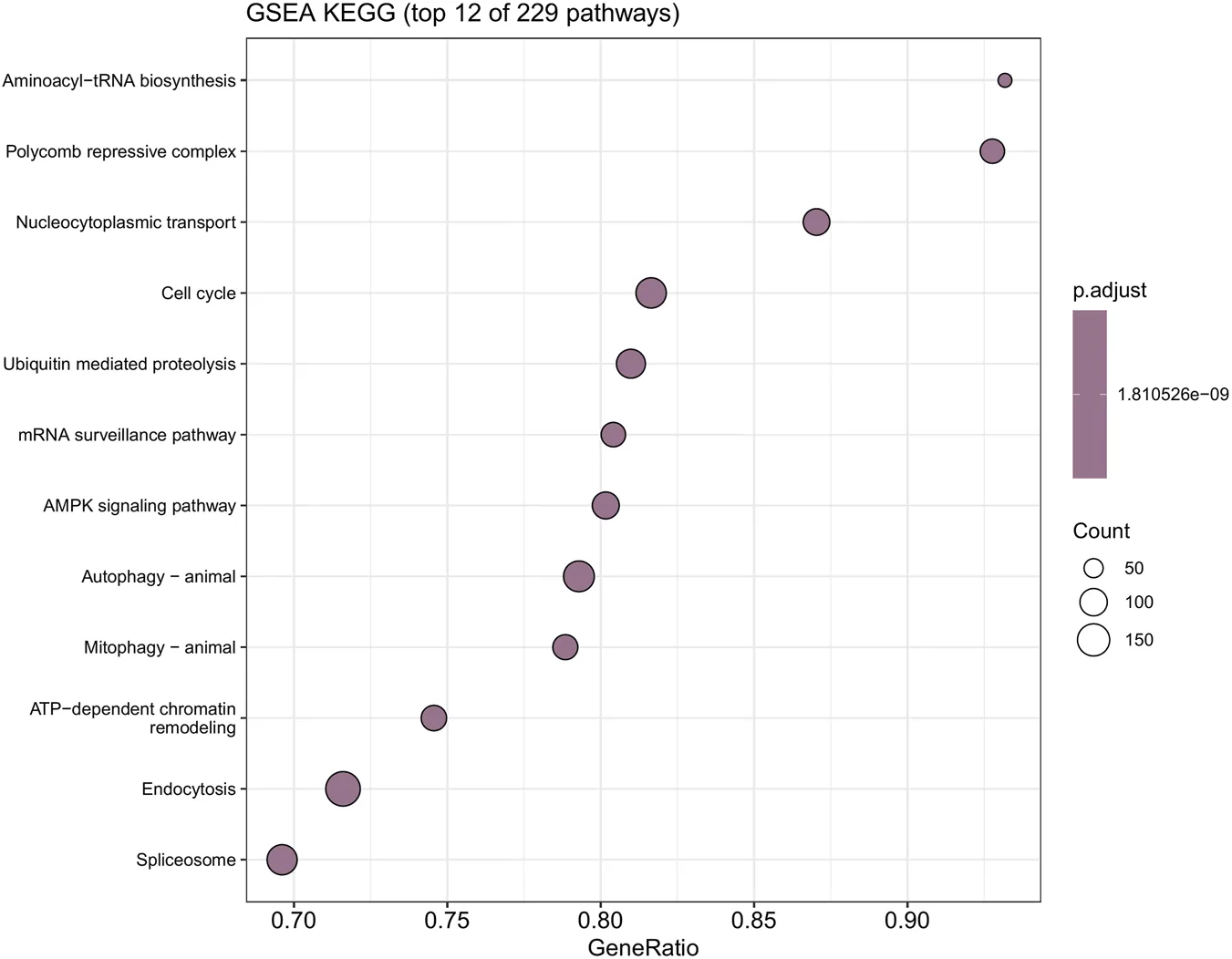
Gene set enrichment analysis (GSEA) against the KEGG pathway database identified 124 significantly enriched terms associated with RNA processing, cell cycle, ubiquitination, and immune signaling pathways (padj < 0.05), ranked by normalized enrichment score (NES). For reproducibility, we fixed the random seed (10000 permutations) to perform analysis on the full ranked gene list. Significant top pathways are shown. The enrichments reflect statistically significant correlations prior to the robustness assessment.

### 3.6 GSEA hallmark enrichment

To investigate the immunoregulatory impact of high DZIP3 expression in GBM, we performed GSEA using the Hallmark molecular signature (padj < 0.05; fixed seed with 10000 permutations to stabilize p-value estimates). We identified 30 significantly enriched gene sets (Supplementary Table 10; Figure 10), mainly associated with cell cycle and mitotic programs (mitotic spindle, G2M checkpoint, MYC targets, E2F targets), metabolic signaling pathways (oxidative phosphorylation, mTORC1signaling pathway, fatty acid metabolism), and developmental signaling pathways (NOTCH, WNT/β-catenin).

**FIGURE 10:**
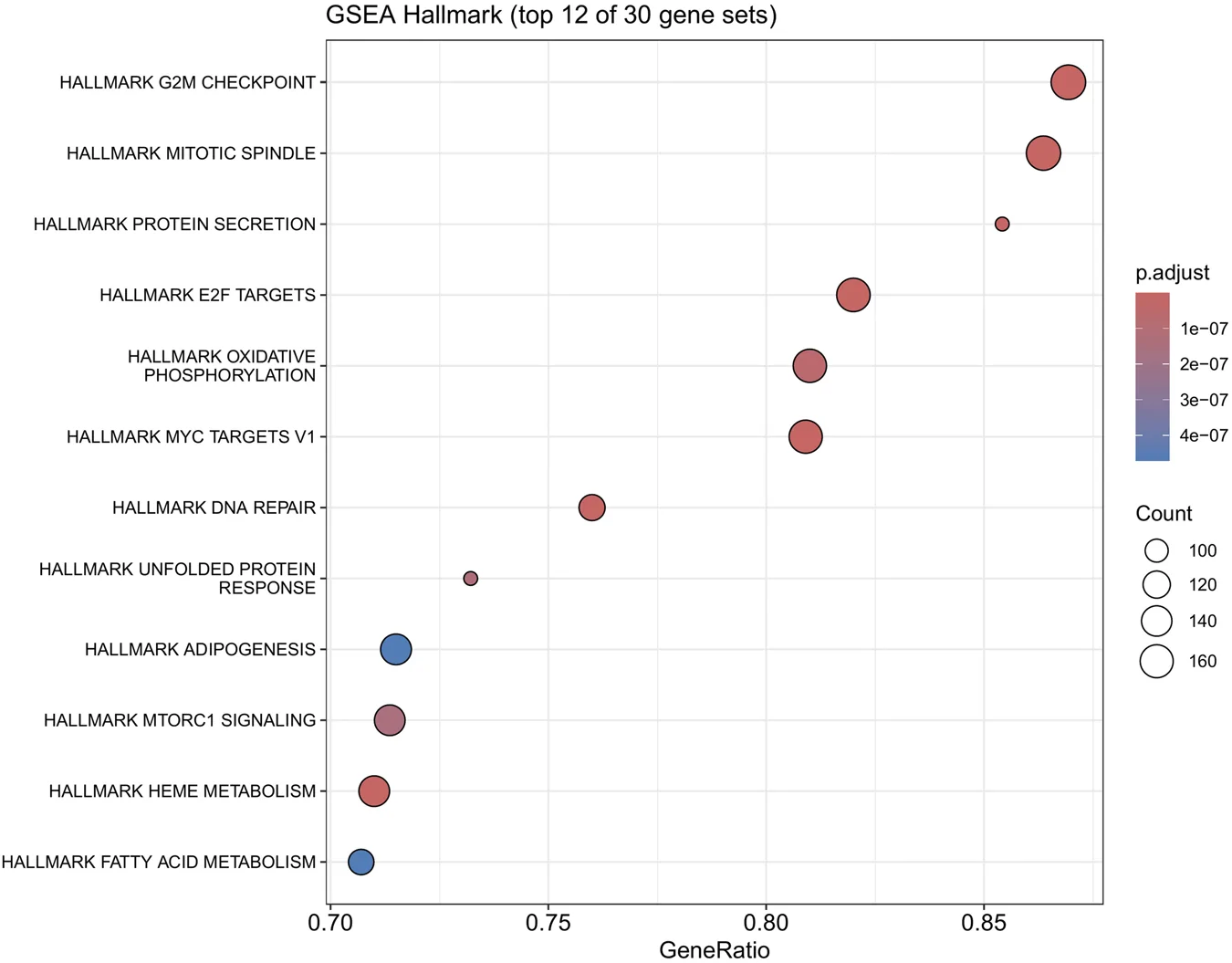
Gene set enrichment analysis against the MSigDB hallmark gene set collection. We identified 30 significantly enriched terms (padj <0.05), ordered by normalized enrichment score. We used a fixed random seed (10000 permutations) to ensure reproducibility.

### 3.7 Immune cellular states infiltrating the TME

Using three complementary algorithms for deconvolution, namely Cibersort (LM22 signature), ImmuCellAI (deconvolution of T cell subpopulations), and a glioma-derived immune signature and malignant cell state classification (Ajaib et al., Neftel et al. signatures, both implemented in the GBMDeconvoluteR package), we examined the immune cell composition and the malignant state of our cohort (Figures 11a–c). We assessed the cell-type-matched concordance between Cibersort and ImmuCellAI across 8 immune populations (Supplementary Table 11). Neutrophils showed a strong correlation (ρ = 0.72, p < 2.2 × 10^−16^), whereas a moderate concordance was observed for total T cells (ρ = 0.30, p = 1.8 × 10^−7^), monocytes (ρ = 0.23, p = 1.2 × 10^−4^), and B cells (ρ = 0.15, p = 0.014). The concordance for dendritic cells and macrophages/tumor-associated macrophages was non-significant (ρ = −0.07, p = 0.25 and ρ = 0.005, p = 0.93, respectively). A significant negative correlation was observed for NK cells (ρ = −0.15, p = 0.011). For Cibersort and the Ajaib signature obtained from GBMDeconvoluteR (Supplementary Table 11), we noticed the same pattern: T cells and monocytes were both concordant (ρ = 0.31, p = 8 × 10^−8^, and ρ = 0.17, p = 0.004, respectively), whereas NK cells, B cells, macrophages, and mast cells were discordant (all p ≥ 0.45). These results corroborate the cell-type-specific discordance among deconvolution algorithms, especially for less abundant immune cell populations. Concordance, when present, is modest in amplitude (p < 0.35). Using the Neftel malignant cell-state classification, we observed that DZIP3 expression was strongly associated with NPC-like and OPC-like states (ρ = 0.73, p < 2.2 × 10^−6^; ρ = 0.69, p < 2.2 × 10^−6^, respectively; Figures 11d,e). The correlation with APC-like and MES-like states was more moderate (ρ = 0.52, p < 2.2 × 10^−6^ and ρ = 0.31, p = 1.5 × 10^−7^, respectively, Supplementary Table 12). After controlling simultaneously for proliferation, immune infiltration, library complexity, and the cytoskeletal compartment, all these associations were eliminated (partial ρ, NPC-like = 0.11, OPC-like = 0.23, AC-like = 0.29, MES-like = 0.08), concordant with the previous patterns observed across all gene sets evaluated in this study.

**FIGURE 11:**
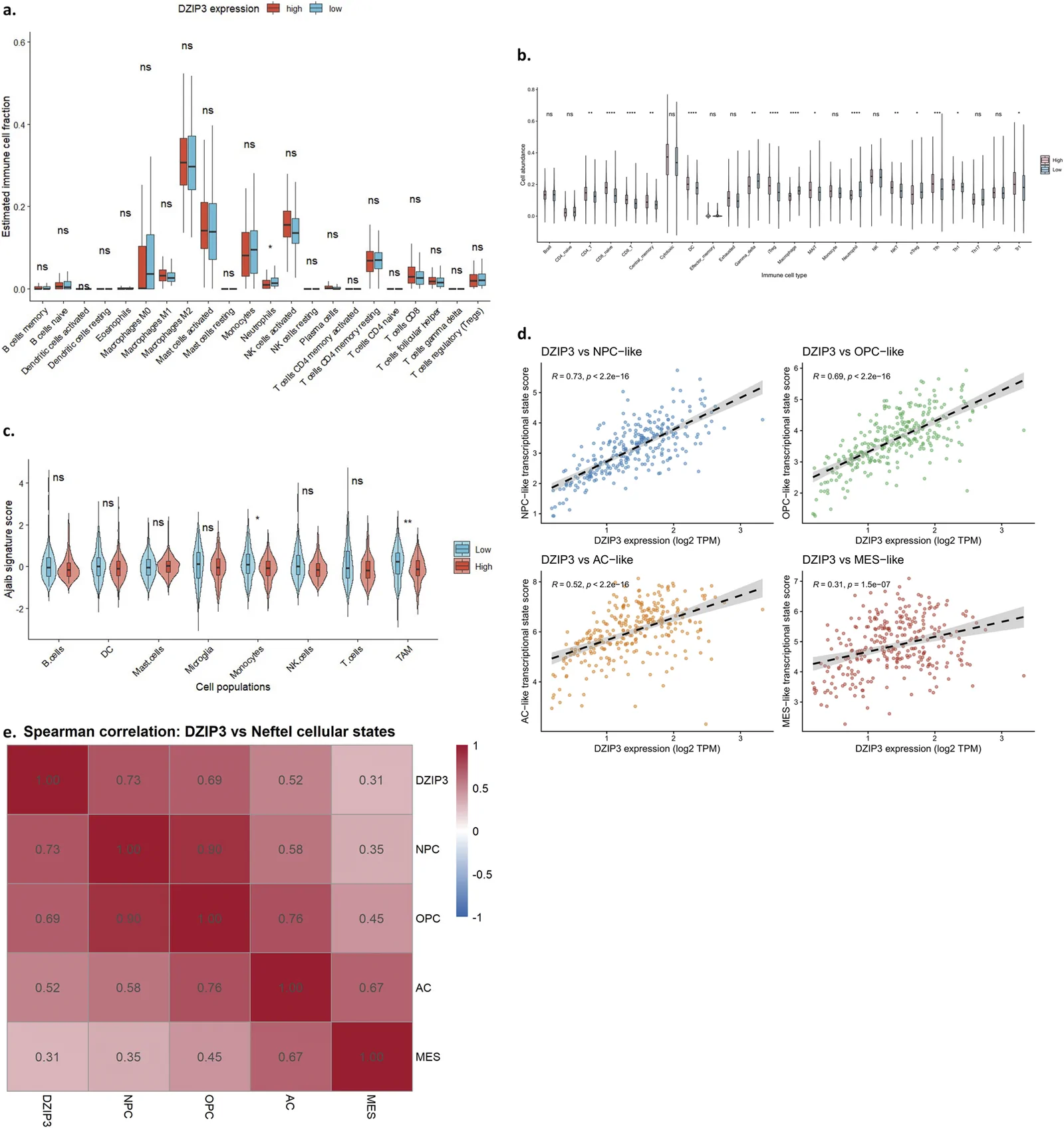
Immune cell deconvolution: **(a)** Immune cell composition between high and low DZIP3 groups using the Cibersort algorithm (n = 287); asterisks are for the Wilcoxon test significance after Benjamini-Hochberg correction (*FDR<0.05, **FDR<0.01, ***FDR<0.001, ns = non-significant; **(b)** Estimated T cell subsets between high and low DZIP3 groups (n = 287) using the ImmuCellAI algorithm; **(c)** Association between DZIP3 expression and immune signatures obtained from the GBMDeconvoluteR algorithm (Ajaib et al.); **(d)** DZIP3 is strongly associated with NPC-like (ρ = 0.73) and OPC-like (ρ = 0.69) states. After robustness assessment, however, the associations were attenuated (partial ρ: NPC-like = 0.11[ns]; OPC-like = 0.23; AC-like = 0.29; MES-like = 0.09 [ns]). Abbreviations: NPC: neural progenitor cell; OPC: oligodendrocyte precursor cell; AC: Astrocyte cell; MES: mesenchymal cell.

### 3.8 DZIP3 expression and inhibitor ICPs

To elucidate DZIP3’s association with the regulation of anti-tumor immune responses, we assessed its correlation with immunosuppressive molecules, namely inhibitory ICPs, which are frequently overexpressed on tumor cells.

In this regard, we noticed a positive correlation between DZIP3 and VTCN1, CD38, BTLA, CD27, LAG3 (CD223), SIRPA (CD172a), PVR, TIGIT, CEACAM1, ADORA2A, CD160, CD47, KLRG1, CD200, ENTPD1, BTN3A1, PTPN2, zymogen granule protein 16 (ZG16), podoplanin (PDPN), HAVCR2, CTLA4 (CD152), PDCD1LG2 (PD-L2), PDCD1 (PD-1), TACTILE (CD96), CD200 receptor 1 (CD200R1), PD-L1 (CD274), TNFRSF5 (CD40), SELPLG (CD162), NRP1 (CD304), Branched Chain Amino Acid Transaminase 1 (BCAT1), KLRC1 (CD159a), TNFRSF14 (HVEM), galectin-9 (LGALS9), Prostacyclin Receptor (PTGIR), Vista (VSIR), CD73 (NT5E), CD6, CD154, CD276 and CD226 (FDR <0.05) (Figures 12a,b). However, based on the correlation score and the p-value, we identified strong positive correlations with CD47, ENTPD1, CD200, PTPN2, BTN3A1, LAG3, SIRPA and CD27, with statistically significant differences (r = 0.67, r = 0.62, r = 0.62, r = 0.58, r = 0.57, r = 0.52, r = 0.51, r = 0.51; p < 0.0001, Supplementary Table 13). Other positive correlations, but not as strong, were assessed with CD226, CD276, CD154, CD6, NT5E, VSIR, PTGIR, LGALS9, TNFRSF14, KLRC1, BCAT1, NRP1, SELPLG, CD40, CD274, CD200R1, CD96, PDCD1, PDCD1LG2, CTLA-4, TIM-3, PDPN, ZG16, and SIRPA. To verify whether these associations are specific to DZIP3 expression, we evaluated a composite checkpoint score (mean log2-TPM across all 49 ICPs) using the previously established robustness framework. The composite score was associated with DZIP3 expression (ρ = 0.42, p = 1.1 × 10^−13^), proliferation (ρ = 0.41, p = 7.5 × 10^−13^), library complexity (ρ = 0.43, p = 1.4 × 10^−14^), and the cytoskeletal dynamics/transport gene set (ρ = 0.34, p = 2.6 × 10^−9^), but not with immune infiltration (ρ = 0.07, p = 0.21). We evaluated the partial correlation controlling for proliferation, immune infiltration, library complexity, and the cytoskeletal gene set. This analysis revealed a reduced association (partial ρ = 0.14, p = 0.015), falling below the FDR-corrected threshold and the pre-specified effect size threshold, indicating that DZIP3 and ICPs expression are not independent of the cytoskeletal transcriptional program identified as the initial correlate of DZIP3 expression in this cohort.

**FIGURE 12:**
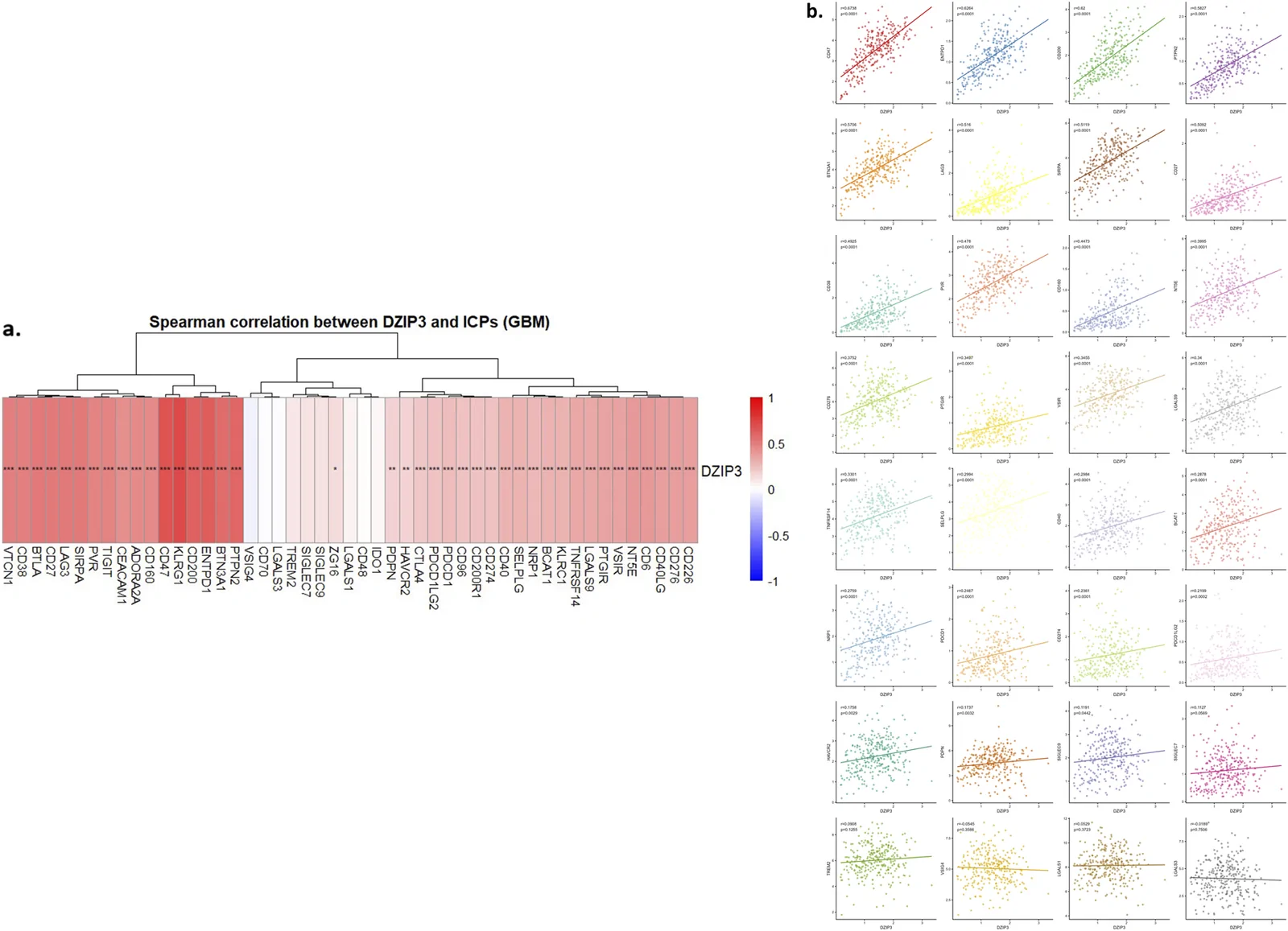
Association between DZIP3 expression and inhibitory immune checkpoint expression (ICP). **(a)** Scatterplot of composite ICP (n = 49) score against DZIP3 expression, with Spearman correlation coefficient and p-value; **(b)** Heatmap representation for individual ICP and DZIP3 expression correlation. After robustness assessment, the correlation was substantially reduced (partial ρ = 0.14), falling below the FDR-corrected significance and effect size thresholds applied in our analysis.

### 3.9 Identification of independent transcriptional signatures through the robustness assessment

Using candidate gene sets obtained from GO, Reactome, KEGG, and Hallmark enrichment analyses, as well as ICPs, GBMdeconvoluteR-derived signatures, and mitochondrial module analyses, and accounting for the risk of confounding from proliferation, immune infiltration, and library complexity, we conducted a robustness assessment. We evaluated candidate gene set scores against DZIP3 expression using Spearman correlation followed by partial Spearman correlation controlling for proliferation, immune infiltration, and library complexity. 30 candidate gene sets were evaluated using Benjamini-Hochberg. They were classified as retained if they respected the threshold that we chose (|partial ρ|≥0.30 and FDR correction (padj <0.05) (Supplementary Table 14); GO-derived terms (P-body elements, chromatin-modifying enzymes, ribosomal proteins, glial/OPC lineage regulators); Reactome signaling pathways (NOTCH and WNT signaling pathways, genome maintenance); KEGG signaling pathways (aminoacyl-tRNA synthetases, ubiquitin-mediated proteolysis, nucleocytoplasmic transport, mRNA decay, TNF signaling, TLR signaling, CXCR signaling, PD-1/PD-L1 signaling pathway), Hallmark-related pathways (mitotic spindle gene set), the panel of 49 inhibitory ICPs, RNA granule-associated genes (TARDBP, FUS, G3BP1/2, HNRNPA1/A2B1, MATR3, ATXN2); signatures obtained from GBMDeconvoluteR (immune signature and NPC-like, OPC-like, AC-like and MES-like malignant cell states) and lastly the ten most strongly correlated mitochondrial functional modules. Among the 30 gene sets, 23 were poorly or not significantly associated with DZIP3 expression after comprehensive confounder control or cross-gene-set control (partial ρ values ranged from 0.02 to 0.44). These gene sets encompassed P-body elements (raw ρ = 0.81, partial ρ = 0.08), chromatin-modifying enzymes (raw ρ = 0.80, partial ρ = 0.08), glial-OPC-like and NPC-like malignant cell states (partial ρ = 0.23 and 0.11, respectively), NOTCH signaling pathway (raw ρ = 0.66, partial ρ = 0.02), WNT signaling pathway (raw ρ = 0.80, partial ρ = 0.1), mitotic spindle gene set (raw ρ = 0.77, partial ρ = 0.15), and the 49-gene ICPs panel. On the other hand, the seven gene sets that met the criteria we fixed earlier are: the cytoskeletal dynamics/intracellular transport gene set (KIF1A/1B/2A/3A/5A/5B/5C/21A/21B, DYNC1H1, MAP1A/1B/2, MAPT, PAFAH1B1, STMN2/4), with a raw ρ = 0.83, partial ρ = 0.51, p = 6.96 × 10^−20^. This gene set shows a stronger association with DZIP3 expression, followed by four mitochondrial modules associated with biogenesis and metabolism (branched-chain amino acid metabolism, partial ρ = 0.44/NAD biosynthesis, partial ρ = 0.41/translation factors, partial ρ = 0.4/pyruvate metabolism, partial ρ = 0.36; padj <10^−8^), and finally two gene sets associated with intracellular transport (nucleocytoplasmic transport, partial ρ = 0.34/RNA granule and neurodegeneration-associated genes, partial ρ = 0.31).

### 3.10 CGGA cohort validation confirms DZIP3 association with the seven gene sets

To confirm the results found in the TCGA cohort, we analyzed CGGA-mRNA-693 for independent validation. DZIP3 was found to correlate with the cytoskeletal dynamics/intracellular transport gene set (ρ = 0.81, p < 2.2 × 10^−16^), corroborating the results from the TCGA cohort (ρ = 0.83, Supplementary Table 15). The other gene sets, including NAD biosynthesis, branched-chain amino acids metabolism, translation factors, pyruvate metabolism, nucleocytoplasmic trafficking, and RNA granule/neurodegenerative gene sets, were all replicated in the CGGA cohort (ρ = 0.58–0.76, Supplementary Table 1). The survival, as mentioned above, was not reproduced in the Chinese cohort.

### 3.11 Validation of the cytoskeletal dynamics/transport signature using single-cell data

We evaluated an independent single-cell RNA sequencing atlas of GBM (Core GBmap; CELLxGENE) to further validate the cytoskeletal dynamics/intracellular transport signature as the strongest correlate of DZIP3 expression in bulk RNA sequencing data (Figure 13). We limited the analysis to malignant cells (n = 127521 of 338564 total cells). DZIP3 expression exhibited a significant positive correlation with the cytoskeletal dynamics/transport gene set (ρ = 0.19, p = 0) at single-cell resolution (Supplementary Table 15). The association remained significant even after controlling for per-cell sequencing depth (partial ρ = 0.11, p = 0), corroborating that the association between DZIP3 expression and this gene set is not an artifact of bulk tissue composition but is evident at the cellular level. Despite this partial correlation not meeting the previously defined effect size threshold due to attenuation of the correlation magnitude at single-cell resolution, the result is consistent with the bulk-related association.

**FIGURE 13:**
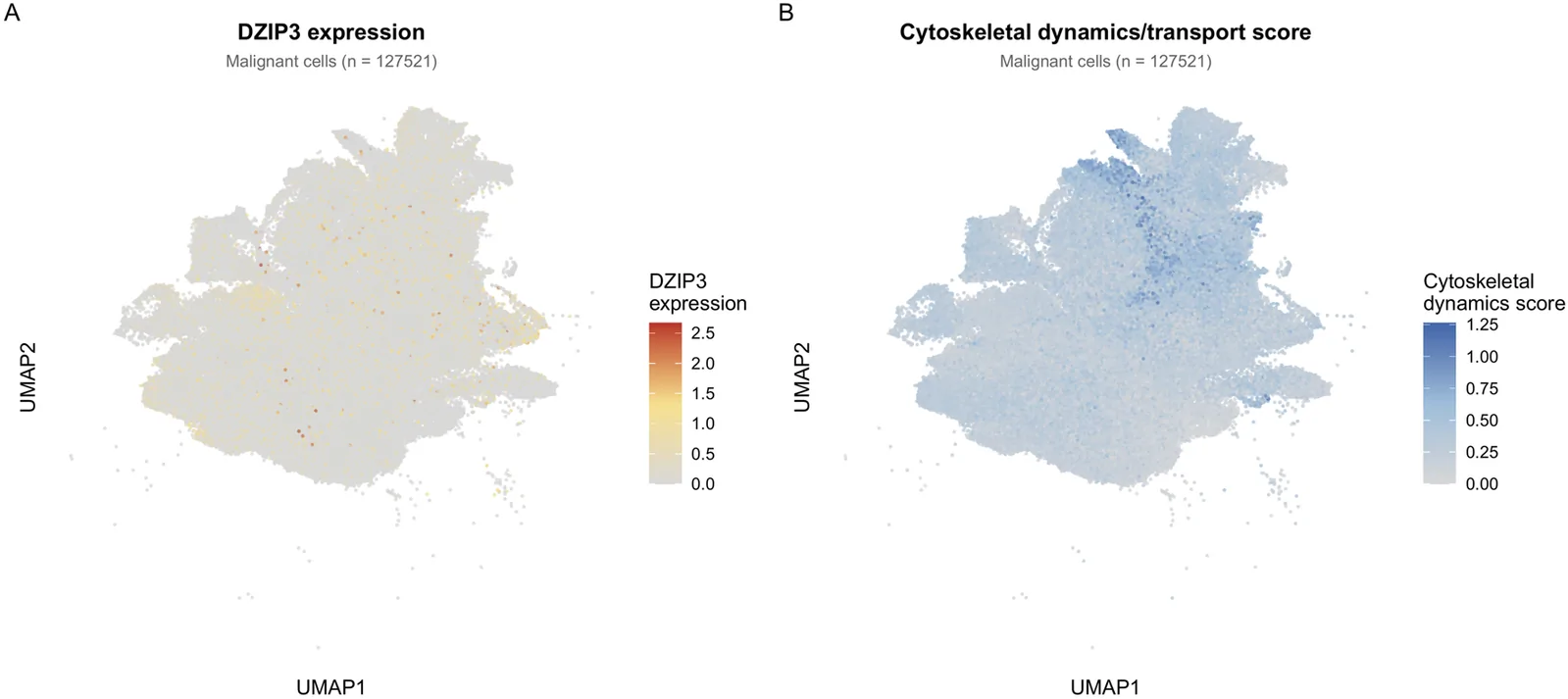
Validation of the cytoskeletal dynamics gene set at single-cell resolution. Using an independent glioblastoma single-cell atlas (Core GBmap, n = 127521 neoplastic cells), we obtained a UMAP projection of malignant cells colored by **(a)** DZIP3 expression and **(b)** cytoskeletal dynamics gene module score (mean expression of cytoskeletal-related genes). The original dataset embedding helped us define the UMAP coordinates. Initially statistically significant and consistent with bulk findings, the association was later attenuated relative to bulk tissue estimates.

### 3.12 DZIP3 is associated with mitochondrial translation and metabolism

To uncover mitochondrial genes associated with high DZIP3 expression, we downloaded data from the MitoCarta3.0 database, which contains 1136 genes. Next, we proceeded to a differential analysis using the Limma package. We then grouped genes into 51 different modules. We established a per-sample score (mean log2-TPM expression) for each module containing 5 or more genes. Next, we correlated the high DZIP3 expression and the per-sample score (Figure 14; Supplementary Table 16).

**FIGURE 14:**
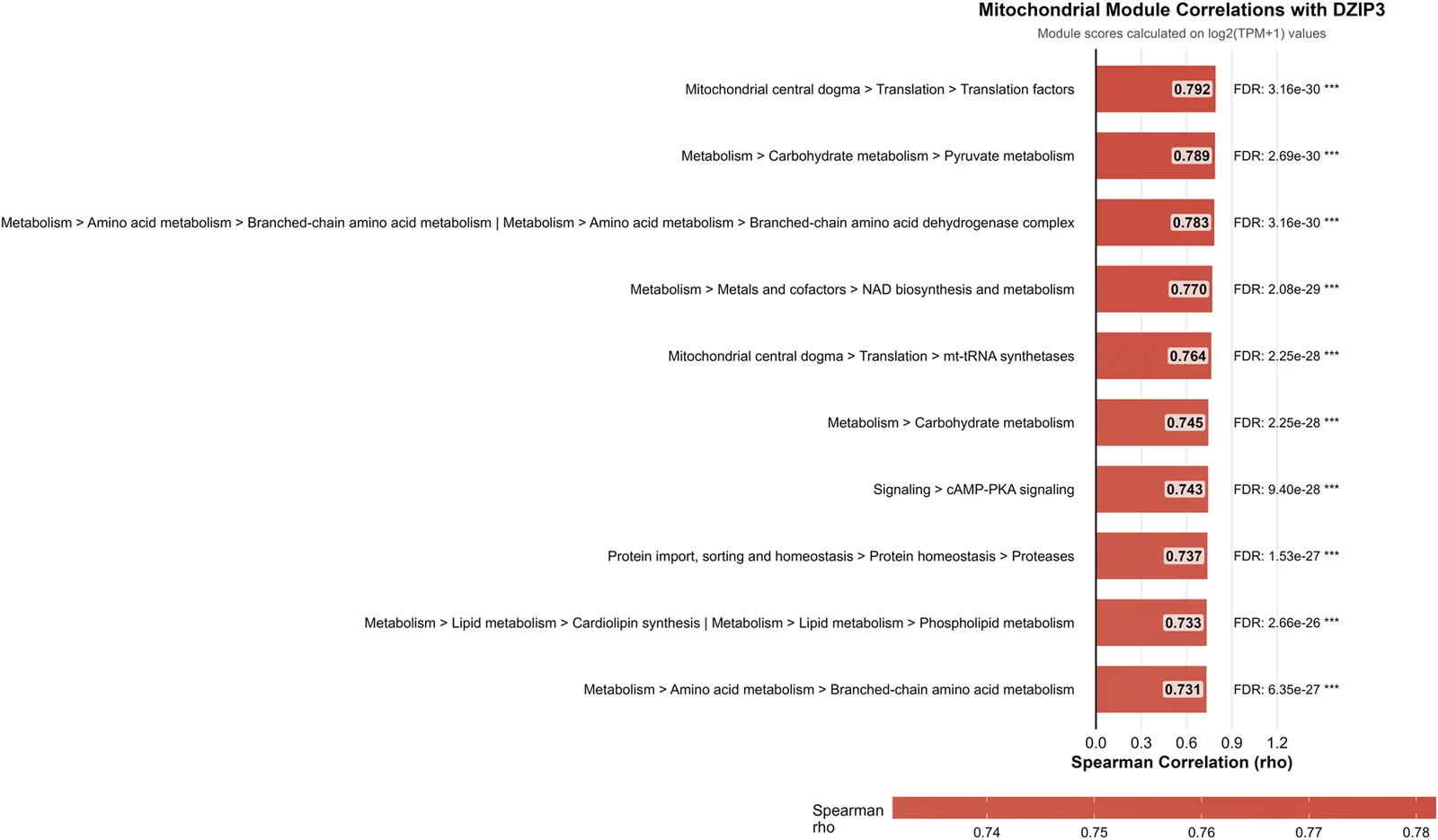
Correlation between DZIP3 expression and mitochondrial modules. We identified the top 10 MitoCarta3.0 modules that are strongly associated with DZIP3 expression. The branched-chain amino acid metabolism, NAD biosynthesis, translation factors, and pyruvate metabolism retained FDR-corrected significance and met the pre-specified effect-size threshold (partial ρ ≥ 0.30) after the robustness assessment.

Ten modules were strongly associated with DZIP3 (ρ = 0.73–0.79). These modules are related to mitochondrial biogenesis, translation machinery, pyruvate metabolism, NAD biosynthesis, and branched-chain amino acid metabolism. Robustness assessment revealed that these associations, although attenuated relative to raw correlations, remained meaningful after controlling for proliferation, immune score, library complexity, and the cytoskeletal component (partial ρ = 0.36–0.44). These clusters, along with the cytoskeletal dynamics/transport gene set, retained independent associations with DZIP3 expression in this study. The branched-chain amino acid metabolism module exhibited the strongest independent correlation (partial ρ = 0.44, p = 1.8 × 10^−14^), indicating its position as the second most robust transcriptional correlate after the cytoskeletal dynamics/transport gene set. Other modules were correlated with DZIP3 expression (NAD biosynthesis, translation factors, and pyruvate metabolism, partial ρ = 0.36–0.41).

### 3.13 mRNA-miRNA network

By quantifying the 1881 miRNA precursors, we identified 776 precursors that mapped to at least one mature miRNA identifier using the multiMir package. Next, we performed differential analysis to identify miRNAs significantly associated with DZIP3 expression (FDR <0.05, |log2FC| > 0.5). We found 49 significant miRNA precursors and 62 differentially expressed mature miRNAs (Supplementary Table 17). By cross-referencing the differentially expressed miRNAs against experimentally validated targets of the 17 genes of the cytoskeletal dynamics/transport gene set, we obtained 51 candidate miRNAs. From these candidates, 9 targeted ≥13 of 17 genes and were identified as core hub regulators (Figure 15; Supplementary Table 18). As such, 7 of these hub miRNAs directly targeted DZIP3, suggesting a possible post-transcriptional regulatory loop controlling DZIP3 expression and its linked cytoskeletal transcriptional program (Supplementary Table 18). The miR-17-92 cluster and its paralogs encompassed 6 of these hub miRNAs (miR-18a-5p, miR-20b-5p, miR-106b-5p, miR-17-5p, miR-20a-5p). The remaining 3 miRNAs are genomically different (miR-27a-3p, miR-185-5p, miR-15a-5p).

**FIGURE 15:**
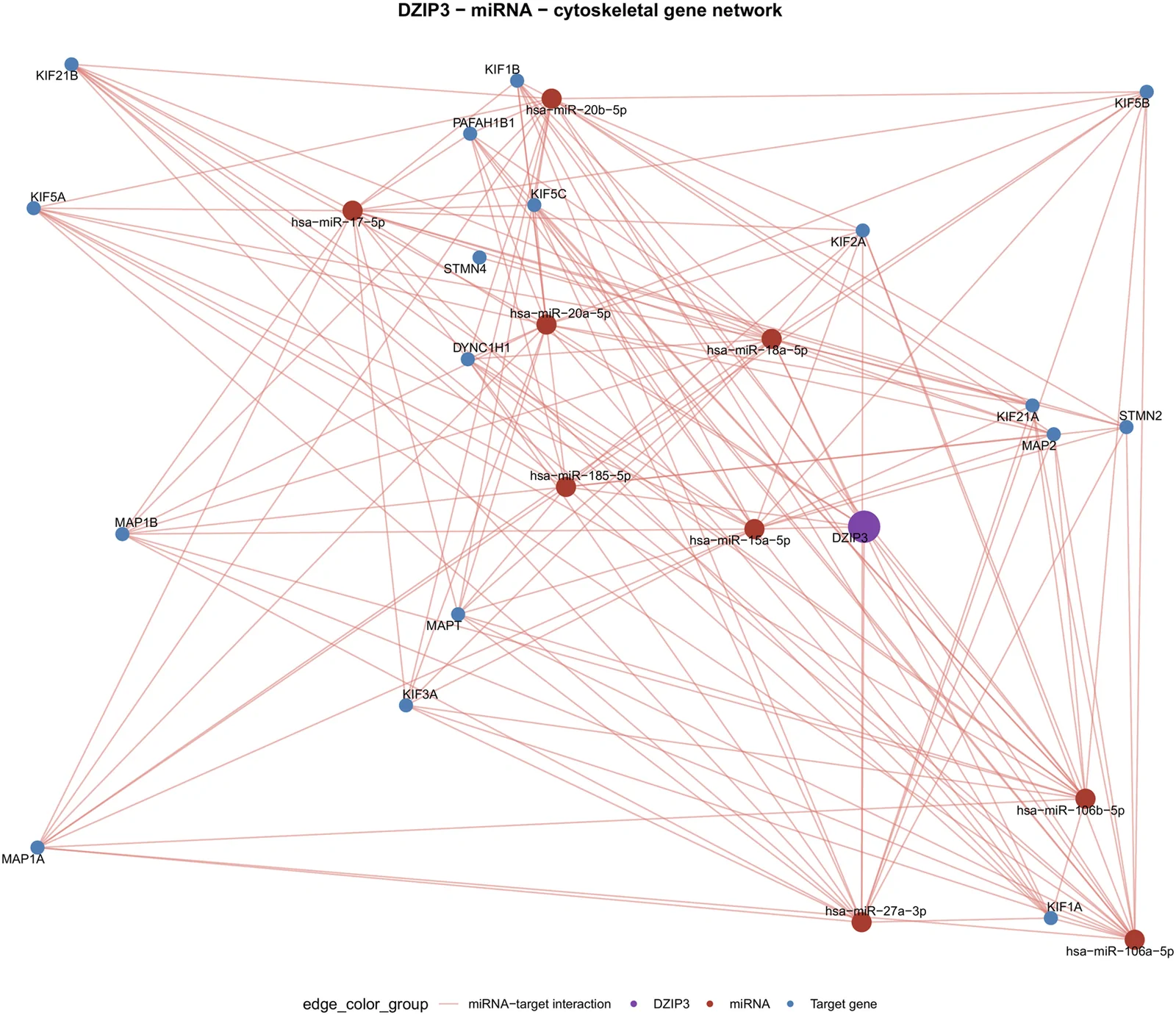
Network associating DZIP3 expression, cytoskeletal dynamics/intracellular transport gene set, and miRNA-mRNA. The network shows core hub miRNAs (n = 9, red nodes) that target ≥13 of the 17 genes in the cytoskeletal dynamics/transport gene set (blue nodes), based on experimentally validated interactions from various databases (miRTarBase, TarBase, miRecords). DZIP3 (purple node) interacts with each hub miRNA via an edge colored according to the direction of the Spearman correlation (green positive, red = negative).

## 4 Discussion

GBM is one of the deadliest tumors occurring in the brain, with a median survival rate of 15–23 months (Alire et al., 2025). The therapeutic resistance to standard treatments could be explained by GBM microenvironmental heterogeneity, in which a dynamic interplay among immune, stromal, and tumor cells is key to cancer progression (White et al., 2024). Moreover, stressors such as variable pH, hypoxia, free radicals, and nutrient deprivation trigger stress responses that enable adaptation to or stress resistance, mainly through SG formation (Attwood et al., 2025). DZIP3, an RBP of SG structures, is the target of our current study.

We performed this study to evaluate the transcriptional and clinical correlates of DZIP3 expression in GBM. We identified numerous candidate gene sets through GO, KEGG, Reactome, and GSEA enrichment analyses, as well as ICP correlations and mitochondrial module analyses. A total of 30 gene sets were retrieved, encompassing P-body elements, chromatin-modifying enzymes, NOTCH and WNT signaling pathways, ICPs expression, NPC-like and OPC-like malignant cell state signatures. All these subsets exhibited strong correlation with DZIP3. However, the robustness assessment, controlling for proliferation, immune infiltration, and technical library complexity, revealed that most of the associations were not independent and converged on 7 gene sets: the cytoskeletal dynamics/intracellular transport gene set, 4 mitochondrial modules (branched-chain amino acid metabolism, NAD biosynthesis, pyruvate metabolism, and translation factors), RNA granule/neurodegeneration gene set, and nucleocytoplasmic transport. After analyzing these gene sets, we found a strong independent association with DZIP3 expression. These correlations persisted even after controlling for confounding factors (partial ρ = 0.51), in the validation cohort (CGGA, ρ = 0-58-0.81), and at single-cell resolution in malignant cells from an independent GBM atlas (GBMCore, partial ρ = 0.11, controlling for sequencing depth). Moreover, at single-cell resolution, the correlation was directionally coherent and statistically significant, despite a reduction in magnitude. Given these results, we suggest that these gene sets are the most robust and biologically meaningful transcriptional correlates of DZIP3 expression identified in this study. Given that DZIP3 is a component of SGs and SGs depend on microtubules, we could hypothesize that DZIP3 expression may be linked to a broader program that harmonizes macromolecular biogenesis and transport, rather than targeting a single pathway. Such a hypothesis requires functional validation.

By performing the robustness assessment, we found that 7 gene sets correlate with DZIP3 expression: the cytoskeletal dynamics/intracellular gene set, the stronger of the correlates, includes kinesin family motor proteins (KIF1A/1B/2A/3A/5A/5B/5C/21A/21B), DYNC1H1, microtubule-associated proteins (MAP1A/1B, MAP2, MAPT), stathmins (STMN2, STMN4), and the dynein regulator PAFAH1B1. The totality of these genes represents the core molecular machinery for microtubule-based intracellular transport and cytoskeletal remodeling. GBM are often described as tumors with a diffuse infiltrative growth pattern. We can then safely suggest that this transcriptional program is associated with invasiveness and cellular motility in GBM. Moreover, these processes are implicated in treatment resistance and the pathobiology of the tumor. Previous studies highlighted the role of the kinesin family in glioma progression (Liu et al., 2025). As such, KIF1AB, KIF1B, KIF2A, KIF3A, and KIF21B have been associated with a worse prognosis in glioma (Chen et al., 2016; Kreft et al., 2014; Yi et al., 2025; Hoang-Minh et al., 2014; Zhang et al., 2016). Other members of the kinesin superfamily are also involved in gliomagenesis. For instance, KIF3C enhances invasion, proliferation, and migration of GBM cells by stimulating epithelial-mesenchymal transition and activating the PI3K/Akt signaling pathway (Gao et al., 2020). Moreover, KIF4A promotes GBM progression by modulating the HIF1A/VEGFA signaling pathway and resistance to temozolomide (Shen et al., 2025). As a matter of fact, dysregulation of cytoskeletal network integrity is associated with reduced invasiveness and motility in GBM, further confirming the importance of this transcriptional program in conferring an invasive phenotype on this tumor (Derby et al., 2024). Altogether, these results point to the direct or indirect involvement of DZIP3, previously identified as an SG component, in a transcriptional program that modulates cytoskeletal organization and intracellular transport in GBM, suggesting that, beyond its translational repression, it may also be associated with motility-related genes. Through our analysis, we identified a mechanistic link between SGs and cytoskeletal dynamics: studies have shown that SG velocity and motility depend on microtubules, and that microtubule depolymerization inhibits SG formation (Nadezhdina et al., 2010). As DZIP3 is a component of SGs, we suggest that the miRNA-mRNA network obtained in our study may indicate that mRNA granule transport, as well as the cytoskeleton, associates SGs-post-transcriptional regulation with the motility-related transcriptional program. In addition to the cytoskeletal dynamics/intracellular transport gene set, we identified 6 additional correlates of DZIP3. The previously retrieved mitochondrial modules, namely branched-chain amino acid metabolism and NAD biosynthesis, are consistent with existing literature reporting that branched-chain amino acid catabolism promotes GBM aggressiveness (Lu et al., 2024). Furthermore, BCAT1 activity is higher in IDH-wild-type gliomas (Tönjes et al., 2013). On the other hand, the study led by Martell et al. demonstrated that increased mitochondrial pyruvate carrier activity and mitochondrial mitotic activity are associated with more aggressive GBM in patients treated with chemotherapy (Martell et al., 2025). Additionally, mitochondrial translation factors are upregulated in GBM upon glucose starvation, thereby promoting GBM cell growth (Miki et al., 2022). Targeting the mitochondrial translation machinery represents an interesting therapeutic approach (Miki et al., 2022). Among the genes associated with the nucleocytoplasmic trafficking gene set is KPNA2, which is associated with inhibited apoptosis and proliferation in GBM, as well as nucleoporins that are correlated with tumor progression across different malignancies (Martinez-Olivera et al., 2018; Huang W. et al., 2025; Nataraj et al., 2022; Holzer et al., 2019; Charles Cano et al., 2025). Among RNA granule-associated genes, G3BP1 is involved in chemotherapy resistance and tumor angiogenesis, and it protects GBM cells from hypoxia-induced death, as it is a core component of SGs (Attwood et al., 2025; Bittencourt et al., 2019). Moreover, TARDBP inhibits stress-induced apoptosis via autophagy, whereas HNRNPA1 is associated with poor survival by upregulating glycolysis-related genes, and HNRNPA2B1 promotes glioma by activating the AKT/STAT3 signaling pathway (Lin et al., 2017; Luan et al., 2015; Yin et al., 2020). The miRNA-mRNA network analysis proposes a candidate regulatory mechanism linking DZIP3 to this program. Indeed, of the nine miRNAs defined as core regulators of the cytoskeletal dynamics/transport gene set, seven directly targeted DZIP3, as reported by experimentally validated interaction databases (Whisnant et al., 2013; Boudreau et al., 2014; Nowakowski et al., 2018). These results encourage us to propose an orchestrated post-transcriptional regulatory circuit in which a common set of miRNAs may mutually inhibit the expression of DZIP3 and, subsequently, its co-regulated cytoskeletal program. This could eventually be consistent with the role of DZIP3 as an SG component, particularly since the latter are the principal sites of miRNA-mediated stalled translation. We stress that this is a correlative, speculative observation: whether DZIP3 is mechanistically implicated in this regulatory circuit or co-regulated in parallel cannot be answered by transcriptomic correlation data alone. The 9 core hub miRNAs obtained in our study are mostly members of the miR-17-92 cluster and its paralogous clusters, miR-106b-25 and miR-106a-363 (Mogilyansky and Rigoutsos, 2013; Zhang et al., 2023; Sperb et al., 2025). These clusters represent the most defined oncogenic miRNA clusters in brain tumors (Zhao et al., 2013; Zhao et al., 2022; Brett et al., 2011). For instance, members of the miR-17-92 cluster were found to disrupt cellular homeostasis in GBM and to increase tumor cell proliferation and reduce apoptotic capacity (Gruszka and Zakrzewska, 2018). Furthermore, individual members of this cluster were found to promote GBM invasion; by activating the WNT signaling pathway, mir-106a enhances the transcription of invasion-related genes, namely MMP2, RUNX2, and NANOG (Li et al., 2016). Similarly, miR-25 promotes GBM proliferation, migration, and invasion by activating the mTOR signaling pathway (Peng et al., 2015). In parallel, miR-20a was found to confer an independent favorable prognosis, illustrating the ability of certain members of this cluster to adapt and assume different functional roles depending on the tumor context (Chocholska et al., 2023). Moreover, miR-15a enhances glioma cell proliferation by mediating epithelial-mesenchymal transition, which is linked to cytoskeletal dynamics (Guo et al., 2021). Additionally, miR-27a-3p, along with miR-24-3p, enhances GBM cell proliferation and invasiveness by upregulating MXI1 (Xu et al., 2013). Collectively, the miRNAs identified in our study and the 7 gene sets that correlate with DZIP3 expression suggest that DZIP3 expression is linked to a harmonized cellular program that integrates intracellular transport and mitochondrial biosynthetic activity. Functional investigation is needed to determine whether this is a stress-adaptive response or a constitutively active state.

Although the other gene sets did not show independence after the robustness assessment, we will briefly discuss them for their biological interest. The P-bodies and chromatin-remodeling enzymes, for example, are aligned with the molecular function of DZIP3 as a component of SGs; their dependence on the cytoskeletal gene set may demonstrate co-regulation. This is further supported by data associating SGs dynamics to microtubule-dependent transport (Nadezhdina et al., 2010). At the same time, the reduced correlation with NPC-like and OPC-like malignant cell states is consistent with findings showing that oligodendrocyte precursor-like glioma cells exhibit cytoskeletal modulation and express migratory gene sets (de Castro et al., 2013). This suggests that these signatures may not be artifacts but may encompass overlapping biology.

In contrast, the ICPs, NOTCH, and WNT correlations did not provide a biological rationale for their attenuation, indicating that the primary correlation was due to shared confounding. These findings refute a genuine co-regulated process and point more toward non-specific correlates of an active transcriptional state in high DZIP3 tumors. Our study contributed significantly to the demonstration that a major part of the transcriptomic correlations reported for DZIP3 expression in bulk RNA-seq data, including ICPs’ expression, chromatin modulation, signaling pathways (NOTCH, WNT), RNA processing, and malignant cell state signatures, point to a single common transcriptomic program. These findings highlight the methodological challenges encountered when working with bulk tumor transcriptomics. Indeed, the confounding effects of cell proliferation, immune infiltration, and the technical library’s complexity lead to covariance among many genes with any gene of interest, regardless of any specific biological relationship. Therefore, the performance of enrichment analyses without controlling for confounding factors is prone to generating apparent diversity of significant biological terms that may, in fact, reflect a single dominant shared theme viewed through different analytical lenses. The robustness assessment framework adopted in this study aims to prioritize biologically meaningful correlations over statistically significant yet nonspecific associations, as in similar bioinformatic studies. Rather than a limitation of the present analysis, the identification of a single robust correlate among 30 candidate gene sets is the main analytical result of this work, directly addressing the concern that bioinformatic studies focusing on correlations overinterpret co-occurring associations as evidence of several independent biological mechanisms. Regarding the prognostic significance of DZIP3 expression, the univariable Kaplan-Meier and Cox analyses indicated that high DZIP3 expression is linked to improved overall survival. However, after accounting for other factors, DZIP3 expression was found to depend on other clinical prognostic factors. In another multivariate model that integrated age, sex, IDH mutation status, MGMT promoter methylation, Karnofsky performance score, and transcriptomic subtype, we found that DZIP3 expression was no longer statistically significant (HR = 1.08, p = 0.66). On the other hand, age, sex, and performance status exhibited their expected associations with outcome. This result was further independently validated in the CGGA cohort, where DZIP3 showed no prognostic correlation after adjustment for age, gender, IDH mutation status, MGMT promoter methylation, and treatment status (HR = 0.87, p = 0.23).

With these findings, we conclude that DZIP3 cannot be considered an independent prognostic biomarker and that its association with ICP expression and malignant cell state signatures is not independent of the cytoskeletal transcriptional program. This conclusion suggests that DZIP3 should not be classified solely as a driver of favorable or aggressive clinical phenotypes. Rather, it is a transcriptional associate of a specific cytoskeletal/motility program, without implications for prognosis.

We recognize several limitations to our work when interpreting these results. Firstly, our study is based solely on *in silico* analysis of publicly available data. External validation using *in vitro*, protein-level, or functional methods was not performed. Therefore, all reported connections, including the cytoskeletal gene set, remain correlational, and the mechanistic involvement of DZIP3 cannot be verified with the data alone. To confirm the hypothesis suggested here, functional validation is required.

Secondly, unsupervised clustering or continuous modeling approaches are more effective than the patient stratification exploited here. Indeed, using median expression dichotomization may not be effective at identifying biologically distinct subgroups.

Thirdly, the deconvolution algorithms revealed variation in immune cell composition, with concordance assessed only on a limited subset of immune cell populations, especially neutrophils. Caution should be exercised when interpreting results for less proliferative populations (B cells, macrophages, dendritic cells, mast cells, NK cells). This discrepancy itself exemplifies the limitation of bulk RNA-seq-based immune profiling.

Fourthly, we did not experimentally test the functional consequence of the miRNA-mRNA network, which was constructed with experimentally validated target databases and differentially expressed miRNAs. Therefore, these results remain purely exploratory and need validation through luciferase reporter assays.

Lastly, despite independent replication of the cytoskeletal dynamics/intracellular transport gene set in the CGGA cohort and at single-cell resolution, and the replication of the correlation of the 6 other gene sets in the CGGA cohort, the non-significant prognostic value of DZIP3 is attributed to reduced sample sizes resulting from data availability constraints after the addition of other clinical covariates. Moreover, although the single-cell data were consistent with the bulk findings, a difference in the correlation magnitude between the two data types was assessed. On the other hand, apart from TPM normalization, additional low-expression gene filtering was limited to removing genes with zero expression across all samples.

### 4.1 Limitations

Several additions could strengthen our study. As such, functional validation through knockdown/overexpression of DZIP3 and assessment of this approach on microtubule reorganization in GBM cell lines would reveal the mechanistic effect of DZIP3, beyond the transcriptomic results reported here. More experiments should be included, namely luciferase reporter assays, to confirm the previously obtained expected miRNA-mRNA regulatory network. Moreover, the lack of replication of DZIP3’s prognostic value in the CGGA cohort warrants further investigation across diverse ethnic cohorts. With the addition of this new data, we could clarify whether DZIP3 expression is a context-dependent prognostic biomarker, as it was not reported in the current study.

## 5 Conclusion

SGs exhibit promising potential to enhance sensitivity to therapy, as targeting them induces cell death (Attwood et al., 2025). Our study revealed that DZIP3 is primarily associated with cytoskeletal and cell motility programs in GBM, as well as mitochondrial biosynthesis and trafficking. This finding was further replicated in the CGGA cohort, while showing no prognostic capacity. Additionally, DZIP3 is associated with 7 hub miRNAs, thereby creating a circuit that may regulate its SG function. Our findings characterize DZIP3 as a transcriptional associate of harmonized intracellular transport and biosynthetic activity. Moreover, it differentiates clinical prognostic value from molecular reproducibility, which mandates further functional investigation to determine whether this correlation reflects a causal regulatory relationship or an adaptation to stress.

## Data Availability

The original contributions presented in the study are included in the article/Supplementary Material, further inquiries can be directed to the corresponding author.

## Supplementary material

SUPPLEMENTARY TABLE 1 Clinical characterization of the TCGA and CGGA cohorts, and comparison between CGGA and TCGA for the correlates.

SUPPLEMENTARY TABLE 2 List of DEGs associated with DZIP3 expression.

SUPPLEMENTARY TABLE 3 Top 20 upregulated genes associated with high DZIP3 expression.

SUPPLEMENTARY TABLE 4 Top 20 downregulated genes associated with DZIP3 expression.

SUPPLEMENTARY TABLE 5 GO:BP pathways enriched in high DZIP3 group.

SUPPLEMENTARY TABLE 6 GO:MF pathways enriched in high DZIP3 group.

SUPPLEMENTARY TABLE 7 GO:CC pathways enriched in high DZIP3 group.

SUPPLEMENTARY TABLE 8 Reactome pathways associated with DZIP3 expression.

SUPPLEMENTARY TABLE 9 KEGG GSEA enriched pathways associated with DZIP3 expression.

SUPPLEMENTARY TABLE 10 GSEA Hallmark enriched pathways associated with DZIP3 expression.

SUPPLEMENTARY TABLE 11 Concordance of immune cell populations between Cibersort, ImmuCellAI and GBMDeconvoluteR.

SUPPLEMENTARY TABLE 12 Association between DZIP3 and malignant cellular states (Neftel scores).

SUPPLEMENTARY TABLE 13 Association between DZIP3 and immune checkpoint expression.

SUPPLEMENTARY TABLE 14 Robustness assessment for the 30 candidate gene sets identified through the different enrichments, immune checkpoint expression, and mitochondrial modules.

SUPPLEMENTARY TABLE 15 Raw and partial correlation in an independent single-cell glioblastoma atlas (Core GBmap).

SUPPLEMENTARY TABLE 16 Correlation between DZIP3 and mitochondrial modules obtained from MitoCarta3.0.

SUPPLEMENTARY TABLE 17 miR-mRNA network with 419 interactions and 51 mature miRNAs.

SUPPLEMENTARY TABLE 18 Final miR-mRNA network with 151 interactions and 9 core hub miRNAs targeting DZIP3 and the cytoskeletal dynamics/intracellular transport gene set.
